# Cisplatin-mediated activation of glucocorticoid receptor induces platinum resistance via MAST1

**DOI:** 10.1038/s41467-021-24845-8

**Published:** 2021-08-16

**Authors:** Chaoyun Pan, JiHoon Kang, Jung Seok Hwang, Jie Li, Austin C. Boese, Xu Wang, Likun Yang, Titus J. Boggon, Georgia Z. Chen, Nabil F. Saba, Dong M. Shin, Kelly R. Magliocca, Lingtao Jin, Sumin Kang

**Affiliations:** 1 Department of Biochemistry and Molecular Biology, Zhongshan School of Medicine, Sun Yat-sen University, Guangzhou, China; 2 Department of Hematology and Medical Oncology, Winship Cancer Institute of Emory, Emory University School of Medicine, Atlanta, GA USA; 3 Department of Pharmacology, Yale University School of Medicine, New Haven, CT USA; 4 Department of Pathology & Laboratory Medicine, Emory University School of Medicine, Atlanta, GA USA; 5 Department of Anatomy and Cell Biology, University of Florida, College of Medicine, Gainesville, FL USA

**Keywords:** Cancer therapeutic resistance, Head and neck cancer, Tumour biomarkers

## Abstract

Agonists of glucocorticoid receptor (GR) are frequently given to cancer patients with platinum-containing chemotherapy to reduce inflammation, but how GR influences tumor growth in response to platinum-based chemotherapy such as cisplatin through inflammation-independent signaling remains largely unclear. Combined genomics and transcription factor profiling reveal that MAST1, a critical platinum resistance factor that reprograms the MAPK pathway, is upregulated upon cisplatin exposure through activated transcription factor GR. Mechanistically, cisplatin binds to C622 in GR and recruits GR to the nucleus for its activation, which induces MAST1 expression and consequently reactivates MEK signaling. GR nuclear translocation and MAST1 upregulation coordinately occur in patient tumors collected after platinum treatment, and align with patient treatment resistance. Co-treatment with dexamethasone and cisplatin restores cisplatin-resistant tumor growth, whereas addition of the MAST1 inhibitor lestaurtinib abrogates tumor growth while preserving the inhibitory effect of dexamethasone on inflammation in vivo. These findings not only provide insights into the underlying mechanism of GR in cisplatin resistance but also offer an effective alternative therapeutic strategy to improve the clinical outcome of patients receiving platinum-based chemotherapy with GR agonists.

## Abstract (web summary)

Glucocorticoid receptor (GR) agonists - used in the treatment of solid malignant tumors to reduce inflammation - could potentially affect the anti-tumor activity of chemotherapy. Here, the authors identify a mechanism of cisplatin resistance observed with GR agonist treatment, and show the binding and activation of GR by cisplatin, which leads to MAST1 activation and subsequent MAPK re-activation.

## Introduction

Platinum-based compounds, such as cisplatin or carboplatin, have been the most active clinical drug class for the treatment of a variety of solid tumors including cancers of the ovary, lung, and head and neck for decades^1–3^. However, significant challenges remain with regard to their activity as it is often accompanied by therapy resistance and side effects. Glucocorticoids are first-line antiemetics that are administered during platinum-based chemotherapy regimens^4,5^. The main actions of glucocorticoids occur through the activation of glucocorticoid receptor (GR), also known as nuclear receptor subfamily 3 group C member 1 (*NR3C1*), which is a ligand-dependent transcription factor belonging to the superfamily of nuclear receptors^6^. GR is typically found in the cytoplasm as a GR-hsp90 heterocomplex, which is assembled by a multiprotein chaperone machinery hsp90/Hop/hsp70/hsp40^7–9^. The steroidal ligand occupying GR in the ligand-binding domain (LBD) recruits GR to the nucleus^8,10^. Multiple factors are involved in nucleocytoplasmic shuttling of the GR. Abelson helper integration site 1 (Ahi1) and tetratricopeptide-repeat (TPR) domain proteins are known to hinder GR nuclear accumulation, while SIRT2 accelerates nuclear translocation of GR by altering hsp90 acetylation that mediates dissociation of hsp90 from the GR complex^11–13^. The nuclear transported homodimer form of GR binds to glucocorticoid response elements (GRE) in the promoters of glucocorticoid responsive genes to activate their transcription^14^. GR induces or represses the transcription of target genes and a series of these target genes are involved in inflammation and immune response^15–17^. Therefore, synthetic derivatives of glucocorticoid such as dexamethasone are widely used for their anti-inflammatory and immune-suppressive properties to relieve symptoms, including hypersensitivity, allergic reactions, edema, nausea, vomiting, and other discomforts that may arise during platinum-based treatment^15,18,19^.

Despite the wide usage of GR agonists for co-medication in cancer chemotherapy as supportive care, a precise role of GR activation in response to platinum-based therapy is largely unknown. Growing evidence suggests that dexamethasone may reduce anti-tumor activity of chemotherapeutic agents including paclitaxel or cisplatin. Previous studies demonstrated that dexamethasone treatment results in blunting cellular senescence, which may be linked with inhibition of NF-κB activity and p53 signaling in cisplatin-treated non-small cell lung carcinoma^20^. Moreover, treatment with a GR agonist enhanced tumor growth and resulted in altered gene expression of anti- and pro-apoptotic factors in a paclitaxel-treated xenograft mouse model of breast cancer^21^. Dexamethasone also attenuated anti-tumor activity of cisplatin and increased expression of adhesion molecules including integrin β1 in ovarian cancer cell lines^22^. Conversely, glucocorticoids had minimal effect on cytotoxicity of cisplatin or paclitaxel in a head and neck cancer cell line UM-SCC-14C, or even enhanced cytotoxicity of cisplatin by suppressing NF-κB activation in the cervical cancer cell line SiHa in vitro^23,24^. Two independent groups have recently demonstrated that the pro-tumorigenic activities including paclitaxel resistance of GR are mediated, at least in part, by the activation of the hippo pathway transducers YAP and TEAD4 in breast cancer^25,26^. However, studies lacked either in vivo validations or clinical correlation assessments, and the detailed mechanisms by which the activation of GR is specifically linked to platinum-based therapy resistance still remain largely elusive.

A group of signaling effectors have been identified as predictive markers of cisplatin resistance. ATPase7A/7B/11B and ERCC1 are involved in pre-target or on-target cisplatin resistance mechanisms, respectively^27–32^. We identified a microtubule-associated serine/threonine kinase 1 (MAST1) as a common essential driver for cisplatin resistance that provides a post-target cisplatin resistance mechanism through managing the pro-apoptotic pathway in human cancers, including lung, ovarian, and head and neck cancers^33^. Mechanistically, MAST1 provides cisplatin resistance by replacing the traditional kinase cRaf when cisplatin disrupts the interaction of MEK and cRaf, which reactivates the MAPK pathway and consequently controls the level of pro-apoptotic factor BIM^33^. Hsp90B was found to stabilize MAST1 by hindering CHIP-mediated ubiquitination at lysine 317 and 545 and preventing proteasomal degradation^34^. In addition, lestaurtinib was identified as a promising MAST1 inhibitor that effectively sensitizes tumor cells to cisplatin in vitro and in vivo in patient-derived xenograft mouse models. However, whether and how MAST1 is induced during treatment for acquired platinum resistance has never been explored.

In this study, through multidisciplinary approaches, we identify a unique GR activation mechanism that is mediated by cisplatin and demonstrate how this activation contributes to cisplatin-resistant tumor growth through MAST1 induction. We also offer a promising therapeutic strategy that may provide an effective regimen for patients who need GR agonists as anti-inflammatory medication along with platinum-based therapy.

## Results

### Platinum treatment induces gene expression of MAST1

**Fig. 1:**
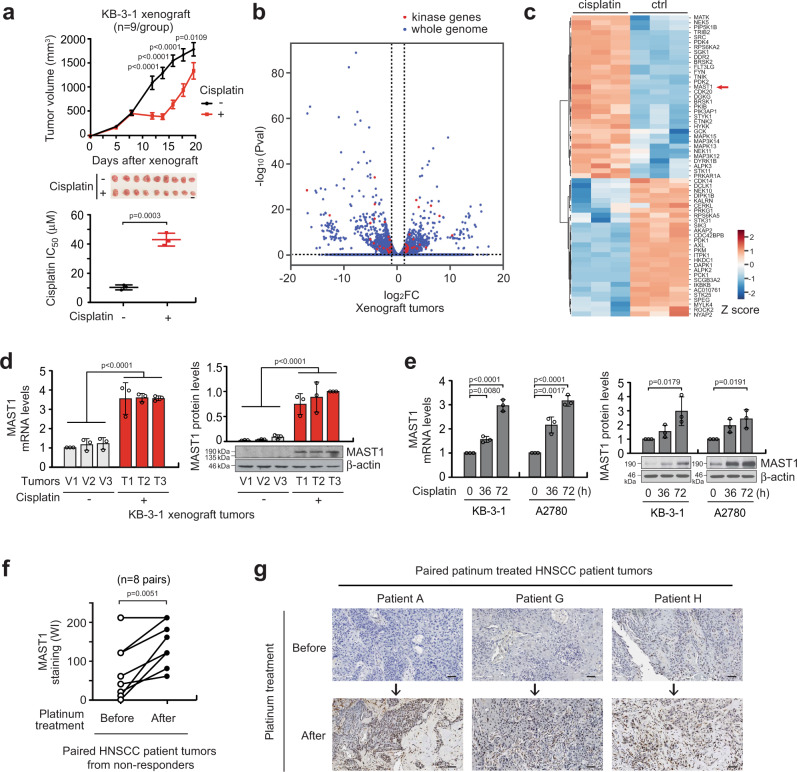
Cisplatin treatment induces MAST1 in preclinical and clinical patient tumors. **a** Development of cisplatin-resistant model in vivo. KB-3-1 xenograft mice were treated with 2 doses of cisplatin (0.5 and 5 mg/kg/i.p. on day 5 and 10). Tumor volumes (top) and cisplatin resistance of three representative xenograft tumors was determined by cisplatin IC_50_ at the experimental endpoint (bottom). **b** Transcriptomic analysis of xenograft tumors collected from mice treated with PBS or cisplatin. RNA-sequencing data are presented as a volcano plot and kinase genes are highlighted in red. **c** A heatmap and hierarchical clustering analysis summarizing expression profile of kinase genes with a fold change greater than 1.2. MAST1 is highlighted with an arrow. **d** Quantitative RT-PCR and western blot results show increased MAST1 expression in KB-3-1 xenograft mice tumors collected from the cisplatin-treated group (T1–T3) compared to the vehicle-treated group (V1–V3). Three representative tumors from each group were randomly selected for analyses. **e** The mRNA and protein levels of MAST1 in KB-3-1 and A2780 cells treated with cisplatin (0.5 μg/ml) for the indicated times were determined as **d**. **f** IHC staining scores of MAST1 in paired primary head and neck squamous cell carcinoma (HSNCC) patient tumors obtained before and after platinum therapy. Patients were considered non-responders to platinum treatment when disease recurred within 2 years after chemotherapy. Weighted index (WI) = positive staining (%) x intensity score (0–3 + ). **g** Representative images of **f** are shown. Scale bars represent 10 mm for **a** and 50 μm for **g**. Data are mean ± SEM for tumor volume in **a** upper panel (*n* = 9 mice/group) and mean ± SD for cisplatin IC_50_ in **a** lower panel (*n* = 3 randomly selected tumors/group). For **d** and **e**, data are mean ± SD from three independent biological experiments. Statistical analyses were performed by two-way ANOVA for tumor volume and unpaired two-tailed *t*-test for IC_50_ in **a** and **d**, one-way ANOVA for **e**, and paired two-tailed *t*-test for **f**. Source data are provided as a Source Data file.

To glean insight into the genetic variation involved in acquired cisplatin resistance, we established an in vivo model of cisplatin-resistant cancer by administering a serial dose of cisplatin to mice bearing KB-3-1 human carcinoma cells (Fig. 1a). Through a whole-transcriptome analysis, we identified a large spectrum of genes that are altered greater than 1.2-fold in tumors that acquired cisplatin resistance compared to treatment-naïve tumors (Fig. 1b). The panel included 61 kinase genes, many of which are important for control of chemotherapy resistant cell growth including previously identified PDK2 and CDK20^35,36^. We identified microtubule-associated serine/threonine kinase 1 (MAST1), as one of the critical cisplatin-induced factors (Fig. 1c). MAST1 confers cisplatin resistance by replacing cRaf to reactivate the MAPK pathway^33^. Induction of the MAST1 gene in xenografted tumors after cisplatin administration in mice was further validated by quantitative RT-PCR and immunoblotting (Fig. 1d). In addition, the expression level of MAST1 was increased upon cisplatin treatment in cancer cell lines including cervical cancer KB-3-1 and ovarian cancer A2780 (Fig. 1e). Consistent with observations in the preclinical setting, comparison of MAST1 expression between paired pre- and post-platinum therapy tumor samples from head and neck squamous cell carcinoma (HNSCC) patients who did not respond to platinum-based chemotherapy further demonstrated that MAST1 expression is induced during treatment (Fig. 1f, g). Non-responders refer to patients with tumor recurrence within 2 years of cisplatin or carboplatin-based chemotherapy. These data indicate that platinum-based drugs such as cisplatin induce gene expression of MAST1 in human cancers.

### GR is a transcription factor that stimulates MAST1 expression upon cisplatin exposure

**Fig. 2:**
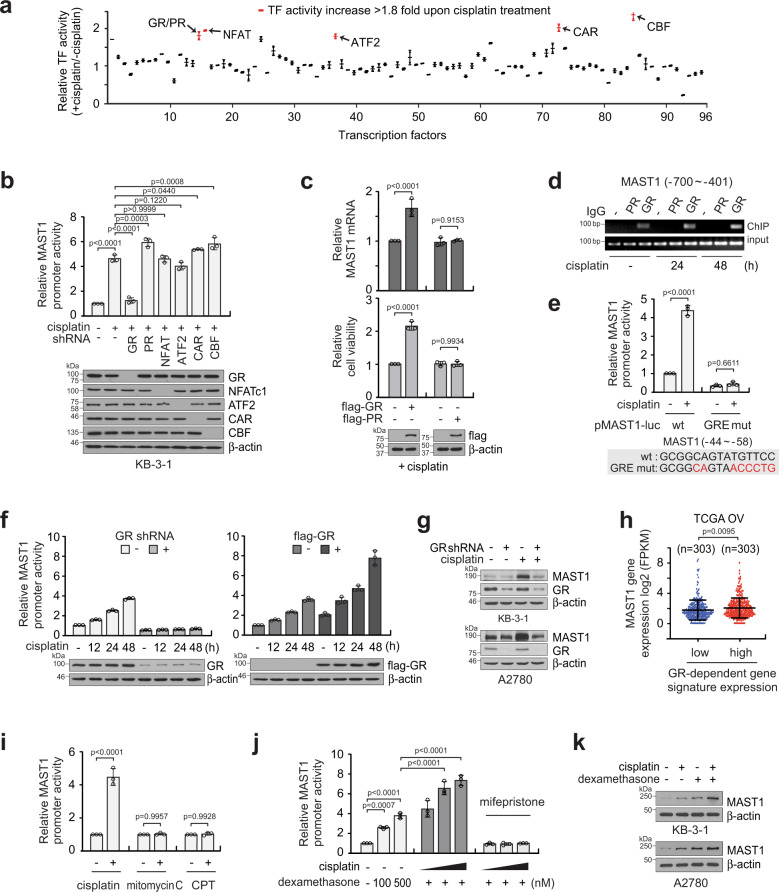
Glucocorticoid receptor (GR), induced by cisplatin, is a transcription factor of MAST1. **a** Transcriptional factor (TF) activation profiling identifies TFs whose activity is enhanced in KB-3-1 cells in response to cisplatin treatment. **b** MAST1 promoter activity in KB-3-1 lacking factors whose activity increased more than 1.8-fold by cisplatin. **c** Effect of GR or PR overexpression on MAST1 induction and cell viability in KB-3-1 cells treated with cisplatin. **d** ChIP assay of GR or PR binding to MAST1 promoter in KB-3-1 cells treated with cisplatin for the indicated times. **e** Comparison of MAST1 promoter activity in cells expressing WT or glucocorticoid response element (GRE)-mutated MAST1 promoter reporter. KB-3-1 cells were transfected with WT or GRE mutant MAST1 promoter reporter and treated with cisplatin. **f** Effect of GR knockdown or overexpression on MAST1 promoter activity in response to cisplatin treatment in KB-3-1 cells. **g** Effect of GR knockdown on MAST1 expression in KB-3-1 and A2780 cells in the presence or absence of cisplatin. **h** Comparison of MAST1 gene expression in TCGA OV patients stratified by GR-dependent gene signature expression monitoring gene expression of 47 glucocorticoid regulated genes. **i** Effect of chemotherapy agents on MAST1 promoter activity. KB-3-1 cells carrying MAST1 promoter were treated with sublethal doses of drugs (1 μg/ml cisplatin, 1 μM mitomycin C or camptothecin). **j** and **k** Induction of MAST1 by cisplatin and dexamethasone. KB-3-1 cells were treated with cisplatin (0.1, 0.2, 0.5 μg/ml), dexamethasone (100, 500 nM), or mifepristone (10 μΜ) for MAST1 promoter activity assay (**j**). GR antagonist mifepristone was used as a control. KB-3-1 and A2780 cells were treated with cisplatin (1 μg/ml), dexamethasone (500 nM), or combination for MAST1 protein induction (**k**). Data are mean ± SD from two technical replicates for **a**, three independent biological experiments for **b**–**g** and **i**–**k**. Statistical analyses were performed by two-tailed *t*-test for **h** and one-way ANOVA for the rest. Source data are provided as a Source Data file.

To glean a comprehensive mechanistic insight into how MAST1 is upregulated upon cisplatin exposure, we performed transcription factor (TF) activation profiling using cisplatin-treated or non-treated KB-3-1 cells. The TF Activation Profiling Plate Arrays monitor activities of 96 cellular TFs, including HIF1, p53, and NF-κB, that are known to be essential in regulating cellular gene expression. Among the 96 TFs, six were activated more than 1.8-fold when cells were treated with cisplatin, including GR/progesterone receptor (PR), NFAT, ATF2, CAR, and CBF (Fig. 2a). To investigate whether any of these TFs activate the MAST1 promoter, we reduced the expression of the 6 potential candidates by shRNA in KB-3-1 cells and performed a MAST1 promoter reporter assay in the presence of cisplatin. Knockdown of GR but no other TFs attenuated MAST1 promoter activity (Fig. 2b). In agreement, overexpression of GR but not PR, which was undetectable in KB-3-1 cells, enhanced MAST1 gene expression and cell viability upon cisplatin treatment (Fig. 2c). Moreover, chromatin immunoprecipitation assay showed that the MAST1 promoter interacts with GR but not PR in KB-3-1 cells and the binding between MAST1 promoter and GR increases upon treatment with cisplatin (Fig. 2d). GR binds to the glucocorticoid response element (GRE) on DNA within the promoter region and regulates genes^37^. We identified a GRE within the MAST1 promoter sequence at −44 to −58. To examine whether GR binds to the GRE in the MAST1 promoter region and enhances MAST1 transcription, we generated a binding-deficient mutant form of GRE in the MAST1 promoter sequence by mutating 6 base pairs of the MAST1 promoter reporter construct, pMAST1-luc. Cisplatin induced WT MAST1 promoter activity. However, when the MAST1 promoter carried the GR binding-deficient mutation in GRE, MAST1 promoter activity was abolished in cancer cells (Fig. 2e). The total amount of MAST1 mRNA increased by cisplatin treatment regardless of promoter activity measured using pMAST1-luc variants (Supplementary Fig. 1a). In addition, modulation of GR affected MAST1 promoter activity in the presence of cisplatin as target downregulation or overexpression of GR, respectively, abolished or enhanced MAST1 promoter activity and mRNA levels in a time-dependent manner (Fig. 2f; Supplementary Fig. 1b). In line with the changes seen in MAST1 mRNA, MAST1 protein level correlated with GR level in cisplatin-treated cancer cells (Fig. 2g; Supplementary Fig. 1c). Collectively, these data indicate that cisplatin results in activation of GR and this activated GR binds to the GRE in the MAST1 promoter region to enhance transcription of MAST1 in cancer cells. Our finding was further validated by analyzing a clinical database. MAST1 levels were higher in ovarian cancer patients with high GR activity compared to patients with low GR activity, which was determined by transcriptome signature of glucocorticoid regulated genes (Fig. 2h). Activation of MAST1 promoter was only observed in cancer cells treated with cisplatin, but not with other DNA damaging agents such as mitomycin C or camptothecin (CPT), demonstrating that the MAST1 induction occurs specifically through cisplatin (Fig. 2i; Supplementary Fig. 1d). Moreover, cisplatin activated the MAST1 promoter, whereas, treatment with the GR agonist dexamethasone additively further enhanced the promoter activity and MAST1 expression (Fig. 2j, k; Supplementary Fig. 1e, f). Moreover, GR agonists including betamethasone, prednisolone, and triamcinolone showed similar induction of MAST1 as dexamethasone (Supplementary Fig. 2a, b). These data suggest that while cisplatin and GR agonists both activate GR, the activation mechanism may differ.

### Cisplatin binds to and nuclear transports GR

**Fig. 3:**
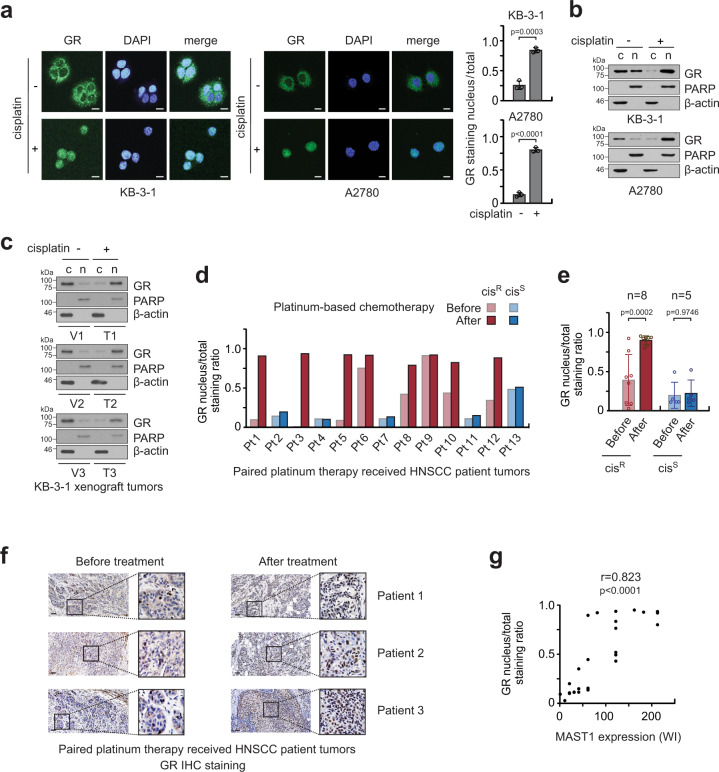
Cisplatin induces GR nuclear translocation. **a** Immunofluorescence assay of GR in KB-3-1 and A2780 cells before and after cisplatin treatment. Nuclei were DAPI stained. **b** Western blots show the cytosolic and nuclear localization of GR upon cisplatin treatment in KB-3-1 and A2780 cells. PARP and β-actin were used as control markers for nucleus and cytosol, respectively. c: cytosol, n: nucleus. **c** GR localization in KB-3-1 xenograft tumors collected from cisplatin-treated (T1–T3) and vehicle-treated (V1–V3) mice. **d** and **e** Nuclear staining of GR in paired HNSCC patient tumor tissues collected before and after platinum treatment. The patients were either “Resistant (cis^R^)” or “Sensitive (cis^S^)” to platinum-based therapy. Images were quantified with ImageJ software. **f** Representative GR staining images of paired HNSCC patient tumors are shown. **g** The correlation between MAST1 expression and GR nuclear staining in paired tumor tissues of HNSCC patients receiving platinum therapy. Scale bars represent 10 μm for **a** and 50 μm for **f**. Data are mean ± SD from three random selected areas for **a**, from one and three biological replicates for **b** and **c**, and *n* = 8 (cisplatin-resistant) and *n* = 5 (cisplatin-sensitive) patients for **e**. Three representative tumors from 13 cases are shown for each group (before/after platinum therapy) for **f**. Statistical analyses were performed by unpaired two-tailed *t*-test for **e** and two-tailed Pearson’s correlation coefficient for **g**. Source data are provided as a Source Data file.

To explore the molecular mechanism by which the transcription factor activity of GR is enhanced by cisplatin treatment, we examined whether cisplatin treatment alters the subcellular localization of GR. We observed that GR is translocated from the cytosol to the nucleus after cisplatin exposure in KB-3-1 and A2780 cells by immunofluorescence staining and cell fractionation (Fig. 3a, b). This observation was confirmed in vivo in xenograft mice. Administration of cisplatin resulted in nuclear translocation of GR in xenograft tumors (Fig. 3c). Furthermore, the translocation of GR was found in paired primary HNSCC patient tumors. HNSCC patients who did not respond to platinum-based therapy and had disease recurrence within 2 years after therapy were considered “cisplatin-resistant.” HNSCC patients who responded to platinum-based therapy by showing no evidence of disease for 2 years after therapy were termed “cisplatin-sensitive.” All patients who received platinum therapy were co-medicated with dexamethasone during treatment. In the case of cisplatin-resistant patients, GR was located mainly in the cytosol in tumors collected from patients before cisplatin or carboplatin therapy, whereas GR was predominantly stained in the nucleus in tumors collected from patients after the therapy. In contrast, the nuclear translocation of GR was not observed in cisplatin-sensitive patient group (Fig. 3d–f). Furthermore, nuclear localization of GR positively correlated with MAST1 expression in HNSCC patient tumors (*r* = 0.823) (Fig. 3g). These data suggest that cisplatin treatment activates GR by translocating it to the nucleus, which may trigger MAST1 expression and acquired platinum resistance in cancer patients.

**Fig. 4:**
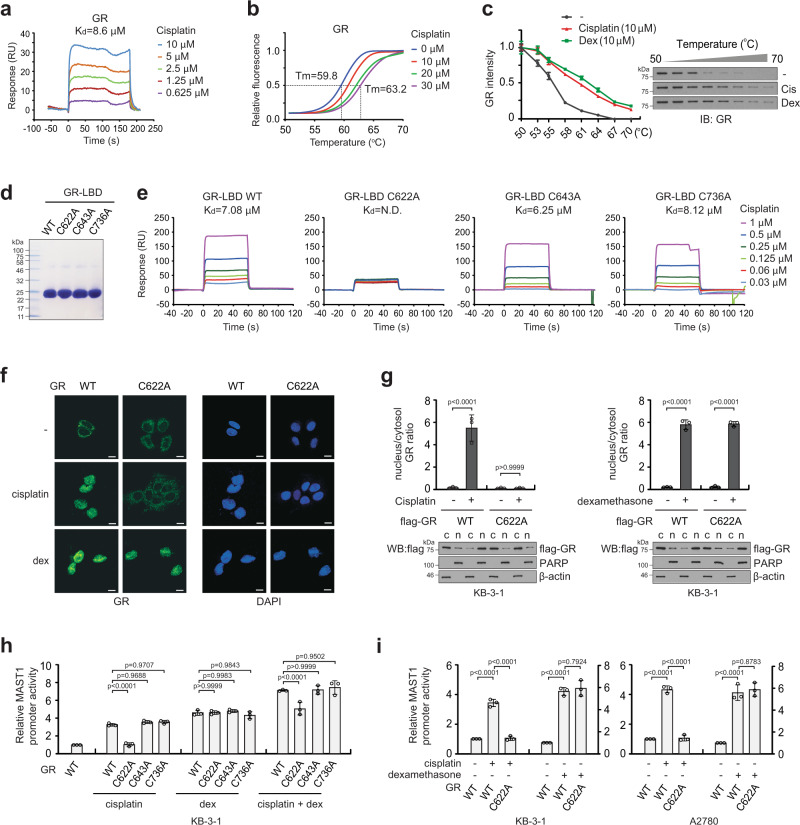
Cisplatin binds to C622 in GR that mediates nuclear translocation and transcriptional activation. **a** Interaction between GR and cisplatin was determined by surface plasmon resonance (SPR) analysis and shown as a dissociation constant value. GR protein was enriched from 239T cells carrying pLHCX-flag-GR. **b** Differential scanning fluorimetry of GR incubated with increasing concentrations of cisplatin. **c** Cellular thermal shift assay using KB-3-1 cells treated with vehicle, cisplatin, or dexamethasone. **d** Coomassie-stained SDS-PAGE gel of purified recombinant GR ligand-binding domain variants, GR-LBDm WT, C622A, C643A, and C736A. **e** Interaction between GR-LBDm variants and cisplatin is determined by SPR analyses. **f**, **g** Effect of cisplatin and dexamethasone on GR nuclear translocation in KB-3-1 expressing GR WT or C622A mutant. KB-3-1 cells were treated with 1 μg/ml cisplatin or 500 nM dexamethasone for 24 h followed by immunofluorescence assay (**f**) or nuclear/cytosol fractionation (**g**). β-actin and PARP were used as cytoplasmic and nuclear markers, respectively. c: cytosol, n: nucleus. Scale bars represent 10 μm. **h**, **i** Effect of cisplatin and dexamethasone on MAST1 promoter activity in KB-3-1 expressing GR WT or CA mutants (**h**) or in KB-3-1 and A2780 cells expressing GR WT or C622A mutant (**i**). Compounds were administered as described in **f**. Data are from one biological experiment for **a**, and mean ± SD from three independent biological experiments for the others. Statistical analyses were performed by one-way ANOVA for **g**–**i**. Source data are provided as a Source Data file.

We next explored the molecular mechanism by which cisplatin treatment translocates GR from the cytosol to the nucleus for activation. Through a series of biomolecular interaction analyses including surface plasmon resonance (SPR), differential scanning fluorimetry (DSF), and cellular thermal shift assay (CTSA), we demonstrated that there is an interaction between cisplatin and GR (Fig. 4a–c). Moreover, the binding affinity of cisplatin to GR was comparable to that of GR agonist dexamethasone (Fig. 4c). To further investigate the interaction between cisplatin and GR, we mutated cysteine residues in the ligand-binding domain (LBD) of GR (Fig. 4d). SPR kinetics analysis using cysteine mutant variants of GR-LBD revealed that WT, C643A, or C736A bound to cisplatin with similar binding strength, whereas C622A mutation abolished the binding ability, suggesting that the C622 residue in GR is responsible for binding to cisplatin (Fig. 4e). Co-immunoprecipitation revealed that cisplatin exposure results in disruption of the GR-hsp90 complex, whereas the dissociation was not observed when GR was mutated at C622 and unable to bind cisplatin (Supplementary Fig. 3). We next investigated the subcellular location of GR WT and C622A in response to cisplatin or dexamethasone treatment by immunofluorescence staining and cell fractionation. While WT GR was translocated from the cytosol to the nucleus upon cisplatin treatment, translocation of C622A GR was not observed. On the contrary, dexamethasone-induced nuclear translocation of GR regardless of C622 mutation (Fig. 4f, g). Treatment with either cisplatin or dexamethasone alone induced MAST1 promoter activity and the combination of these two drugs resulted in additive enhancement of MAST1 induction. However, mutation at C622 abolished the MAST1 promoter activation and expression induced by cisplatin but had no effect on MAST1 induction mediated by GR agonists, including dexamethasone, betamethasone, prednisolone, and triamcinolone (Fig. 4h, i; Supplementary Fig. 4a–c). These data suggest that GR undergoes dissociation from hsp90 and nuclear translocation following cisplatin binding at C622 that consequently induces MAST1 transcription through GRE in the MAST1 promoter region. Although both cisplatin and GR agonists activate GR via interaction and nuclear translocation, the interaction of cisplatin is independent of the interaction and nuclear translocation of GR mediated by the GR agonists.

### GR provides cisplatin resistance to cancer cells through MAST1

**Fig. 5:**
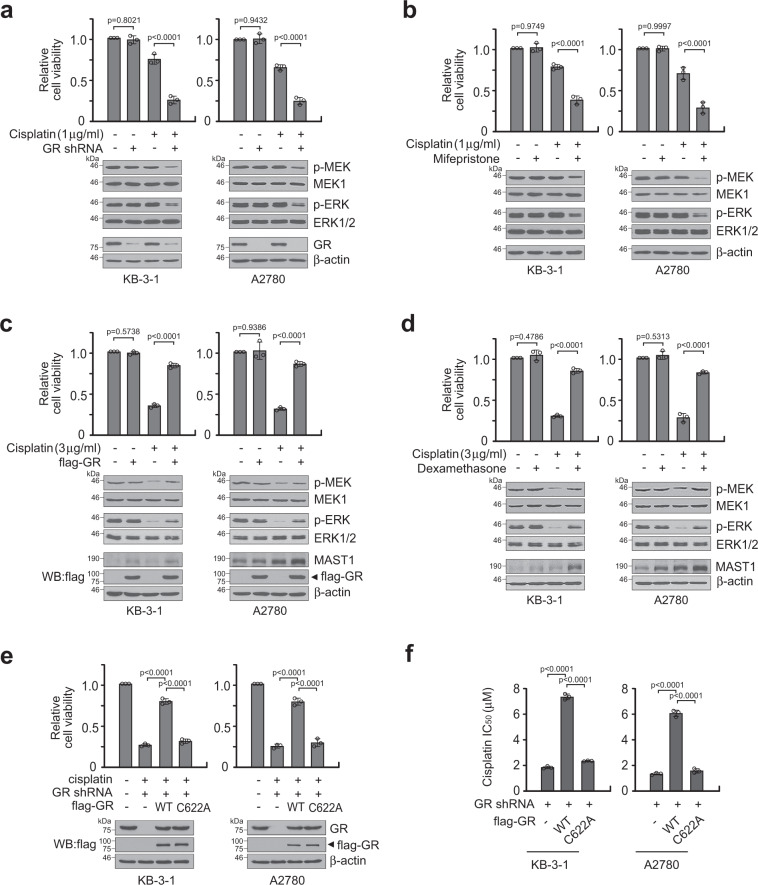
GR C622 contributes to cisplatin resistance through MAST1. **a**, **b** Cell viability and MEK/ERK activity in GR target downregulated KB-3-1 and A2780 cells by GR shRNA (**a**) or mifepristone (10 μM) (**b**) in the presence and absence of cisplatin. MEK and ERK activity was assessed by S221 MEK and T202/Y204 ERK phosphorylation. **c**, **d** Cell viability and MEK/ERK in GR enhanced KB-3-1 and A2780 cells by GR overexpression (**c**) or dexamethasone (500 nM) (**d**) in the presence and absence of cisplatin. **e** Cell viability of KB-3-1 and A2780 cells carrying GR WT or C622A in response to cisplatin. **f** Cisplatin IC_50_ of KB-3-1 and A2780 cells harboring GR WT or C622A. Data are mean ± SD from three independent biological experiments. Error bars represent SD and statistical analyses were performed by one-way ANOVA. Source data are provided as a Source Data file.

**Fig. 6:**
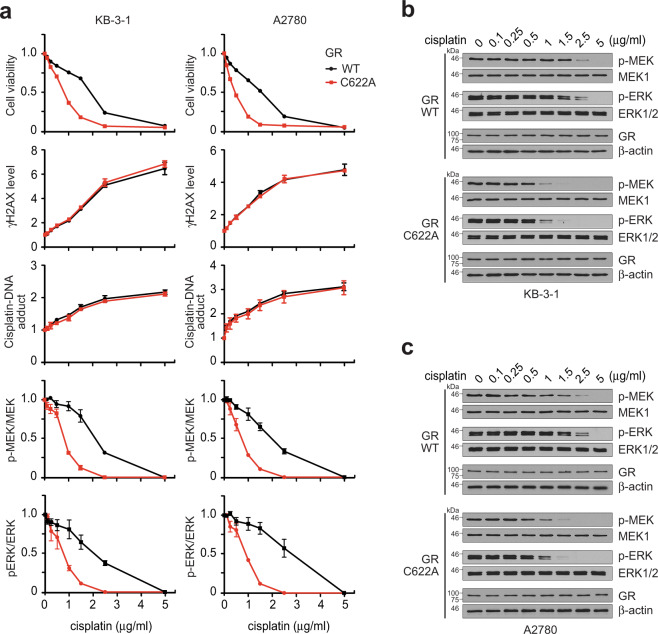
GR C622 is crucial for cisplatin resistance through MEK-ERK activation but not DNA damage response. **a** Dose-dependent analyses of cell viability, γH2AX, cisplatin-DNA adduct, MEK and ERK phosphorylation in KB-3-1 and A2780 cells carrying GR WT or C622A upon cisplatin treatment. **b**, **c** Representative Western blot of MEK-ERK pathway change in KB-3-1 (**b**) and A2780 (**c**) cells carrying GR WT or C622A upon treatment with increasing concentrations of cisplatin. Data are from three independent experiments and mean ± SEM for blot analyses and mean ± SD for others. Source data are provided as a Source Data file.

We previously reported that MAST1 confers cisplatin resistance by replacing cRaf and reactivating MEK signaling^33^. To explore the role of GR in cisplatin resistance and MAPK pathway activation, we target downregulated GR using lentiviral shRNA vector or a GR antagonist mifepristone in KB-3-1 and A2780 cells. While genetic and pharmacological inhibition of GR did not alter cancer cell proliferation and MEK-ERK activation, GR inhibition significantly decreased cell viability and MEK-ERK activity when the cells were treated with a sublethal dose of cisplatin (Fig. 5a, b; Supplementary Fig. 5a, b). We then assessed the impact of enforced GR expression or activation on cisplatin resistance and MEK-ERK signaling in KB-3-1 and A2780 cells. Enforced GR expression or activation by retroviral vector bearing flag-GR or GR agonists significantly enhanced the cisplatin-resistant cell survival potential and MAPK signaling activity (Fig. 5c, d; Supplementary Fig. 5c–e). The effect of GR rescue expression on restoring cisplatin resistance was abolished when GR harbored C622A, the cisplatin-binding-deficient mutant that lacks the ability to induce MAST1 expression (Fig. 5e, f). To further explore the cisplatin resistance mechanism mediated by GR C622, we assessed cell viability, cisplatin uptake, cisplatin-induced DNA damage, and MEK-ERK activity in cancer cells bearing GR WT or C622A mutant treated with increasing concentrations of cisplatin. Mutation at C622 of GR attenuated cisplatin-resistant cell survival and MEK-ERK activity. However, cisplatin uptake or cisplatin-induced DNA damage was not altered in cells bearing C622A GR compared to cells with WT GR (Fig. 6a–c). These data suggest that GR may confer cisplatin resistance through interaction with cisplatin that mediates MAPK pathway activation.

**Fig. 7:**
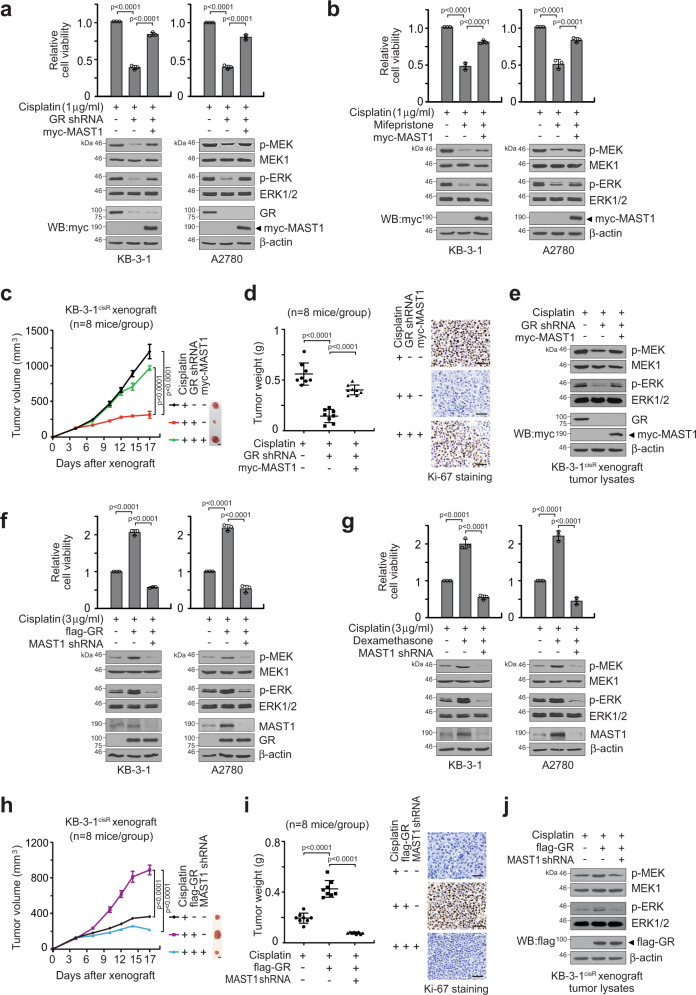
GR promotes cisplatin-resistant cell survival and tumor growth via MAST1-MEK-ERK. **a**, **b** Effect of MAST1 overexpression on cell viability and MEK/ERK activity in GR attenuated KB-3-1 and A2780 cells with cisplatin treatment. GR was target downregulated by GR shRNA (**a**) or mifepristone (10 μM) (**b**). MEK and ERK activity was assessed by S221 MEK and T202/Y204 ERK phosphorylation. **c**–**e** Effect of MAST1 overexpression on tumor growth of xenograft mice bearing GR knockdown KB-3-1 cells. Mice were treated with cisplatin (2 mg/kg i.p. twice/week) from 4 days after xenograft. Tumor volume (**c**), tumor weight and Ki-67 staining (**d**), and phosphorylation of MEK and ERK in tumors (e) are shown. **f**, **g** Effect of MAST1 knockdown on cell viability and MEK/ERK activity in GR enhanced KB-3-1 and A2780 cells with cisplatin treatment. GR was enhanced by GR overexpression (**f**) or dexamethasone (500 nM) (**g**). **h**–**j** Effect of MAST1 knockdown on tumor growth of xenograft mice bearing GR overexpressed KB-3-1 cells. Mice were treated with cisplatin (4 mg/kg i.p. twice/week) from 4 days after xenograft. Tumor volume (**h**), tumor weight and Ki-67 IHC staining (**i**), and MEK and ERK activity in tumors (**j**) are shown. Scale bars represent 5 mm for (**c**, **h**), and 50 μm for (**d**, **i**). Data are from three independent biological experiments for (**a**, **b**, **f**, **g**) and *n* = 8 mice/group for **c**–**e** and **h**–**j**. One representative data are shown for (right panels of **d** and **i**; **e** and **j**). Data are mean ± SD for (**a**, **b**, **d**, **f**, **g**, **i**) and mean ± SEM for **c** and **h**. Statistical analyses were performed by two-way ANOVA for **c** and **h**, and one-way ANOVA for others. Source data are provided as a Source Data file.

To further investigate whether GR confers cisplatin resistance through MAST1, we tested whether ectopic overexpression of MAST1 can reverse the attenuated cell viability and MAPK activity in GR target downregulated cells. Stable overexpression of active MAST1 rescued the decreased cisplatin-resistant cell proliferation and MEK-ERK phosphorylation in cells with GR shRNA or mifepristone (Fig. 7a, b; Supplementary Fig. 6a, b). Similar results were obtained in vivo when MAST1 was overexpressed in GR knockdown tumors in xenograft mice (Fig. 7c–e; Supplementary Fig. 6c). On the contrary, knockdown of MAST1 abolished cisplatin-resistant cancer cell proliferation, tumor growth, and MEK-ERK phosphorylation obtained by enforced GR expression or activation in vitro (Fig. 7f, g; Supplementary Fig. 6d, e) and in vivo (Fig. 7h–j; Supplementary Fig. 6f). In all, 2 mg/kg and 4 mg/kg of cisplatin was administered to adequately study the effect of GR-MAST1 modulation on cisplatin sensitization and resistance, respectively. These data collectively suggest that GR confers cisplatin resistance by inducing the gene expression of MAST1.

### Lestaurtinib abolishes cisplatin and dexamethasone-induced cisplatin resistance

**Fig. 8:**
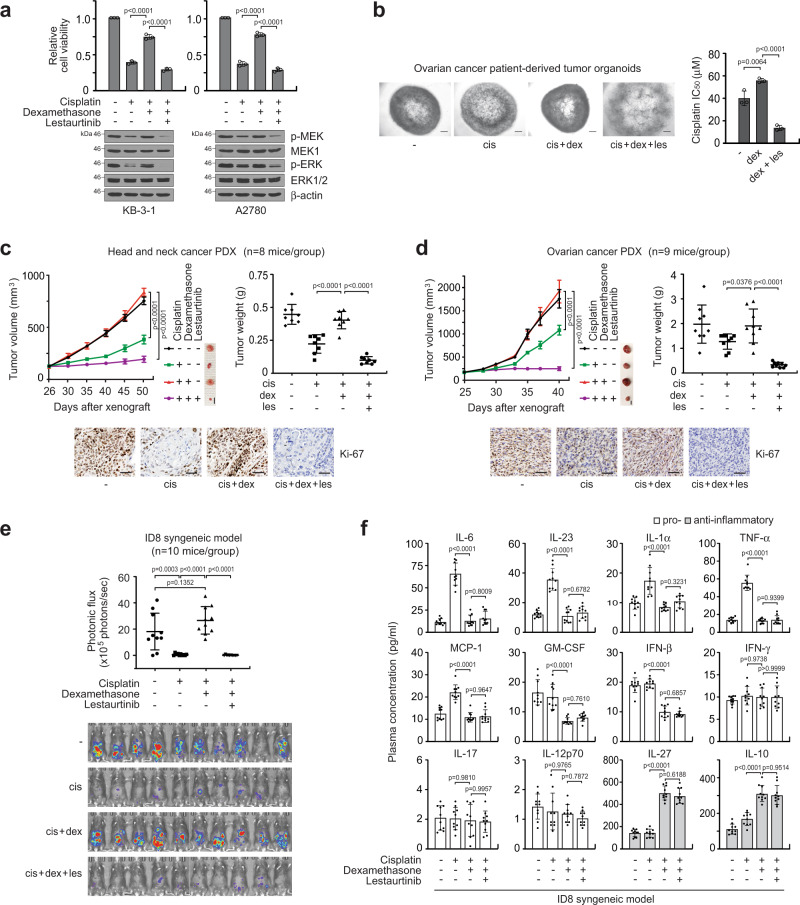
Lestaurtinib suppresses dexamethasone-mediated GR-MAST1 activation and cisplatin resistance while not affecting the anti-inflammatory property of dexamethasone. **a** Effect of lestaurtinib on cell viability and MAPK activity of dexamethasone and cisplatin co-treated KB-3-1 and A2780 cells. Cells were treated with 100 nM lestaurtinib, 500 nM dexamethasone, and 3 μg/ml cisplatin. **b** Effect of lestaurtinib on growth of ovarian cancer patient-derived xenograft tumor organoid treated with cisplatin and dexamethasone. Images (left) and cisplatin IC_50_ (right) of organoids are shown. Single cell suspended organoids were used for IC_50_ analysis. Scale bars represent 100 μm. **c**, **d** Effect of lestaurtinib on tumor growth of HNSCC (**c**) and ovarian cancer (**d**) PDX mice receiving cisplatin and dexamethasone treatment. Cisplatin (5 mg/kg i.p. twice/week), dexamethasone (0.1 mg/kg i.p. twice/week) and lestaurtinib (20 mg/kg s.c. daily) were treated. Tumor volume (left), tumor weight (right), and Ki-67 IHC staining of PDX tumors (bottom) are shown for HNSCC and ovarian PDX models. Scale bars represent 5 mm for tumor images and 50 μm for Ki-67 staining. **e**, **f** Effect of lestaurtinib on tumor growth and inflammation of ID8-luc syngeneic mice receiving cisplatin and dexamethasone treatment. Syngeneic mice were treated as PDX mice. Average photonic flux and BLI (**e**) and plasma inflammation related cytokine profile (**f**) from 10 mice/group at week 8 are shown. Data are mean ± SD from three independent biological replicates for **a** and **b**. In vivo data are from 8 mice for **c**, 9 mice for **d**, 10 mice for **e** and **f**. Data are mean ± SEM for tumor volume (left panels in **c** and **d**) and SD for tumor weight (right panels in **c** and **d**), photonic flux (**e**), and cytokine profiling (**f**). Statistical analyses were performed by two-way ANOVA for tumor volume and one-way ANOVA for the rest. Source data are provided as a Source Data file.

Our findings indicate that GR agonists that are often given with platinum-based chemotherapy, promote acquired cisplatin resistance by enhancing MAST1 expression. Thus, we examined the effect of targeting MAST1 with a small molecule inhibitor lestaurtinib on cisplatin re-sensitization in cancer cell lines and patient-derived tumor organoids treated with GR agonists. Dexamethasone, betamethasone, prednisolone, or triamcinolone restored cisplatin-resistant cancer cell survival and MEK-ERK phosphorylation, whereas treatment with the MAST1 inhibitor diminished the elevated MEK-ERK activity and cisplatin resistance in GR agonist-treated tumor cells (Fig. 8a, b; Supplementary Figs. 7a–c and 8a). We next evaluated the effect of lestaurtinib on dexamethasone-induced cisplatin-resistant tumor growth in vivo in patient-derived xenograft (PDX) mouse models of head and neck cancer and ovarian cancer. Administration of dexamethasone abolished the effect of cisplatin on tumor growth and proliferation, whereas treatment with lestaurtinib in the dexamethasone-treated group fully revived cisplatin sensitivity and even further attenuated tumor growth compared to the group treated with cisplatin alone in both PDX models bearing tumors derived from head and neck cancer or ovarian cancer patients (Fig. 8c, d; Supplementary Fig. 8b, c). Known alternative lestaurtinib targets including Trk and FLT3 were not expressed in tumor cells we studied (Supplementary Fig. 9a). In addition, the inhibitory effect of lestaurtinib on dexamethasone-induced cisplatin-resistant cell growth was abolished in cells are lacking MAST1 but not JAK2. (Supplementary Fig. 9b–d). These data suggest that lestaurtinib provides cisplatin sensitivity by inhibiting MAST1. Lastly, to address the effects of lestaurtinib on the anti-inflammatory activity of dexamethasone during chemotherapy, we established the ID8-luc syngeneic mouse model. C57BL/6 mice treated with dexamethasone and cisplatin displayed greater tumor growth than mice that received vehicle or cisplatin. In agreement with observations in PDX models, lestaurtinib significantly abolished tumor growth potential in dexamethasone-treated mice (Fig. 8e). Quantification of 12 representative pro- and anti-inflammatory mouse cytokines in plasma revealed that dexamethasone effectively decreased pro-inflammatory cytokines, including IL-6, IL-23, IL-1α, TNF-α, MCP-1, GM-CSF, IFN-β and elevated secretion of anti-inflammatory cytokines IL-27 and IL-10. However, lestaurtinib did not influence cytokine release or modulation mediated by dexamethasone (Fig. 8f). These data collectively show that lestaurtinib effectively attenuates cisplatin-resistant tumor growth that is induced by dexamethasone without affecting the anti-inflammatory activity of dexamethasone in vivo.

## Discussion

While platinum-based chemotherapy is the standard front-line therapy for patients with solid malignant tumors, treatment is often accompanied by toxic side effects and thus glucocorticoids are widely used as co-medication to reduce inflammation and relieve symptoms. Despite the conventional usage of GR agonists as antiemetic regimens for chemotherapy, how GR signaling cross-talks with platinum resistance in human cancer has not been fully characterized. Here, we delineate a unique molecular mechanism by which GR is activated by cisplatin and steroid, and how the activation of GR contributes to platinum resistance in human cancers through MAST1 using a series of preclinical studies and analysis of platinum-treated patient tumor specimens. We found that GR is activated upon cisplatin treatment by cisplatin binding to GR and transporting GR from the cytosol to the nucleus. Further activation of GR occurs by its agonists such as dexamethasone in a cisplatin-independent manner. The activated GR contributes to cisplatin resistance by serving as a transcription factor of MAST1, which consequently reactivates MEK. We also clinically validated our findings by demonstrating a correlation between the nuclear localization of GR and MAST1 expression levels as well as cisplatin resistance in primary tumor tissue samples collected from cancer patients before and after treatment with platinum-containing regimens.

We demonstrated that cisplatin induces GR activation by binding to GR at C622, which leads to MAST1 expression and phosphorylation of MEK and ERK. Cisplatin mediates GR nuclear translocation and consequently promotes GR activity as a transcription factor. We previously demonstrated that cisplatin also binds to MEK1 at C142 that mediates disruption of the MEK1-cRaf complex allowing MAST1 to serve as a substitute for cRaf in MAPK signaling^33^. During these processes, the tethering of cisplatin to GR or MEK did not alter its uptake or its ability to induce DNA damage or repair, suggesting that cisplatin has a dual effect in cancer cells. In addition to its conventional role in DNA crosslinking, cisplatin may serve as a signaling mediator in cancer cells to either translocate or dissociate protein-protein interactions that are critical in post-target mechanisms of resistance. In support, other studies report interactions between cisplatin and cellular factors^38,39^.

Our study revealed that cisplatin binds to GR at cysteine 622. Mutation at C622 abolished the interaction between cisplatin and GR and retained GR in the cytosol, which impaired the induction of MAST1 transcription and cisplatin resistance. However, C622A mutation did not alter the GR agonist-mediated nuclear translocation and transcriptional activity of GR. Therefore, cisplatin and GR agonists such as dexamethasone may not share the same GR interaction and activation mechanism in cells. Dexamethasone is a larger moiety that binds to a canonical deep pocket in GR^40^, whereas, cisplatin is a smaller molecule that attaches to the cysteine residue, and these may lead to different binding states. GR forms a multiprotein complex in cells with hsp90 and others^7–9^. It is possible that cisplatin integrated into GR alters the shape of the complex or stabilizes a state that hsp90 cannot bind. We found that MAST1 is protected from proteasomal degradation by binding to hsp90^34^. However, hsp90β but not hsp90α induced MAST1 stabilization whereas both equivalently have chaperone activities on GR, suggesting a differential involvement of hsp90 isoforms in MAST1 stability and GR activation process^34,41^. Future studies are warranted to define the structural basis by which cisplatin and steroids coordinate GR activation.

We demonstrated that MAST1 expression and stability are enhanced by GR and hsp90β, respectively, upon cisplatin exposure. Levels of MAST1 increased by GR and maintained by hsp90β-mediated protection against degradation may lead to a consequent surge in activated MAST1. It is plausible that MAST1 is further activated or inhibited via its phospho-dynamics through cellular kinases or phosphatases in cancer cells. For instance, potential MAST1 interacting partners from the STRING database including membrane-associated guanylate kinase MAGI-2 or serine/threonine-protein phosphatase PPP2R2C/B could be alternative MAST1 regulators that drive cancer cisplatin resistance.

Ectopic expression of MAST1 fully restored cisplatin resistance lost by GR attenuation. In line with this result, genetic or pharmacological enhancement of GR did not offer cisplatin resistance to cancer cells when MAST1 was absent. These studies indicate that although GR transcriptionally regulates a variety of genes, GR mainly programs cisplatin-resistant pro-survival signaling in human cancers through transcriptional regulation of MAST1.

GR signaling is engaged in a variety of functions in malignant cells. In lymphocytes, GR is known to arrest cell growth and induce apoptosis, thus glucocorticoids are considered effective anti-cancer agents for lymphatic cancers^42^. However, GR is known to play a contrasting role in solid malignant tumors. For instance, a high level of GR was linked with differential enhancement of epithelial-mesenchymal transition and cell adhesion in breast cancer^43^. A recent study demonstrated that GR increased tumor heterogeneity and metastasis through ROR1 in breast cancer^44^. Moreover, activation of GR caused increased proliferation and invasion in metastatic colon cancer through CDK1^45^. In our study, we provide clinical evidence that GR nuclear translocation positively correlates with MAST1 expression and platinum resistance in head and neck cancer patients. This suggests that the GR-MAST1 signaling axis could be a promising predictive marker for acquired platinum resistance in human cancer. In addition, cisplatin but not other chemotherapeutic agents such as mitomycin C or camptothecin induced GR activation in cancer cells, suggesting that the signaling axis may serve as an effective biomarker specifically for patients receiving platinum-based therapy.

Lastly, pharmacological studies using PDX and syngeneic mouse models indicate that co-treatment with GR agonist dexamethasone and cisplatin fosters tumor growth. This suggests that the frequent use of steroids as a component of supportive oncology, namely as a major element of the cisplatin antiemetic regimen, may need to be re-examined. Targeting MAST1 using a well-tolerated small molecule kinase inhibitor lestaurtinib may be a method to abrogate the possible effects of steroids on tumor growth as treatment with lestaurtinib effectively re-sensitized tumors to cisplatin treatment while not interrupting the anti-inflammatory activity. Although further optimization of treatment conditions in the clinic is warranted, our study provides a mechanism by which GR agonists along with platinum drugs induce resistance and demonstrate that use of a MAST1 inhibitor may provide more effective platinum-based chemotherapy regimens for patients receiving a GR agonist such as dexamethasone as an anti-inflammatory drug.

## Methods

### Reagents

Human lentiviral short hairpin RNA (shRNA) clones targeting GR, PR, NFAT, ATF2, CAR, CBF, JAK2, and MAST1 were obtained from Open Biosystems. Human GR, GR-LBD, PR, and MAST1 were flag or myc tagged by PCR and subcloned into pLHCX-derived or pET53-DEST Gateway destination vectors as previously described^46^. MAST1 promoter reporter was obtained from Switchgear Genomics. MAST1 GRE mutant promoter reporter and GR-LBDm (GR-LBD-F602S/A605V/V702A/E705G/M752T^47^) and its cysteine mutant variants were generated using site-directed mutagenesis reagents from Agilent. Primer sequences are listed in Supplementary Table 1. CellTiter-Glo Luminescent Viability assay and LightSwitch Luciferase Assay were obtained from Promega and Active Motif, respectively. TF Activation Profiling Array was purchased from Signosis. The LEGENDplex Mouse Inflammation Panel was obtained from Biolegend. Cisplatin, dexamethasone, betamethasone, prednisolone, triamcinolone, and mifepristone were purchased from Sigma-Aldrich. Mitomycin and camptothecin were from Selleckchem. Lestaurtinib was obtained from Tocris Bioscience. A2780 and ID8 cells were purchased from Sigma-Aldrich. KB-3-1 cells were obtained as previously described^48^. KB-3-1 is a derivative of HeLa commonly used in multiple drug resistance studies^49^. 293T cells were from American Type Culture Collection. All cell lines were authenticated by STR profiling. Ovarian PDX tumor for organoid culture was purchased from Jackson Laboratory.

### Antibodies

Antibodies against GR (12041/D6H2L), PR (3153/C89F7), phospho-MEK1/2 (S221) (2338/166F8), MEK1/2 (9126/47E6), phospho-ERK1/2 (T202/Y204) (4376/20G11), ERK1/2 (4695/137F5), myc (2276/9B11), hsp90 (4877/C45G5), phospho-histone H2A.X (S139) (9718/20E3), PARP (9532/46D11), pan-Trk (92991/A7H6R), and Jak2 (3230/D2E12) were obtained from Cell Signaling Technology. MAST1 antibodies (NBP2-17228 and NBP1-81453) were obtained from Novus Biologicals. Antibodies against FLAG (F7425 and F1804/M2) and β-actin (A1978/AC-15) were purchased from Sigma-Aldrich. Antibodies against cisplatin-modified DNA (ab103261/CP9/19) and Ki-67 (ab92742/EPR3610) were obtained from Abcam. Anti-ATF-2 antibody (sc-242/F2BR-1) and anti-FLT3 (sc-479/C-20) antibody were from Santa Cruz Biotechnology. Antibodies against CEBPZ (31-163) and NFATC1 (MA3-024/7A6) were purchased from ProSci and Invitrogen, respectively. Anti-CAR/NR1I3 antibody (PP-N4111-00/N4111) was obtained from R&D Systems.

### Cell and organoid culture

A2780 cells were cultured in RPMI 1640 medium with 10% FBS. KB-3-1, 293T, and ID8 cells were cultured in Dulbecco Modified Eagle Medium (DMEM) with 10% FBS. Lentivirus production, infection, and stable cell selection for gene knockdown or overexpression and protein overexpression were previously described^46,50^. Cells were treated for 48 h with 1 μg/ml and 3 μg/ml cisplatin to study the effect of GR-MAST1 modulation on cisplatin sensitization and resistance, respectively, unless specified. For organoid culture, ovarian PDX tumors were minced into 1 mm diameter pieces and crushed. The homogenates were digested with 2.5 mg/ml Type II collagenase in basal culture media [advanced DMEM/F12 medium supplemented with 1x GlutaMAX, 1% HEPES, and 1% penicillin–streptomycin] for 25 min at 37 °C. The cell pellets were washed twice with red blood cell lysis buffer and resuspended in 75% Matrigel at a concentration of 1 × 10^6^/ml. Approximately 1.5 × 10^4^ cells were loaded into each well of a 48-well plate and cultured in general culture medium [advanced DMEM/F12 medium supplemented with 100 ng/ml R-spondin 1, 100 ng/ml Noggin, 50 ng/ml, EGF, 10 ng/ml FGF-10, 10 ng/ml FGF2, 2% B-27, 10 mmol/l nicotinamide, 1.25 mmol/l N-acetylcysteine, 1 μmol/l prostaglandin E2,10 μmol/l SB202190, 500 nmol/l A83-01, and 10 μmol/l Y-27632]^51,52^. Organoids were single cell suspended and cell viability was measured using CellTiter-Glo Luminescent Viability Assay^33^.

### Transcriptome profiling and quantitative RT-PCR

RNA was isolated using RNeasy Mini Kit (Qiagen) from tumors collected from KB-3-1 xenograft mice treated or non-treated with cisplatin. Poly-A RNA-sequencing was conducted by LC Sciences according to the manufacturer’s instruction. Quantitative RT-PCR was conducted with High-Capacity cDNA Reverse Transcription Kit (Applied Biosystems), Universal SYBR Green Supermix (Bio-Rad), and primers designed for MAST1 using PrimerBank.

### Cell viability assay and cisplatin sensitivity analysis

Cells were seeded on 96-well plates one day prior to the addition of compounds with indicated concentrations for 48 h. Cell viability was measured using CellTiter-Glo Luminescent Viability Assay. Half-maximal inhibitory concentration (IC_50_) values were obtained using GraphPad Prism 8 to measure cisplatin sensitivity.

### Transcription factor activation profiling

KB-3-1 cells were treated with 5 μg/ml cisplatin for 24 h and nuclear proteins were extracted. The activities of 96 essential transcription factors were monitored by TF Activation Profiling Plate Array II (Signosis) according to the manufacturer’s protocol^53,54^.

### ChIP and promoter reporter assay

ChIP assays were performed using chromatin immunoprecipitation assay (Millipore). In brief, endogenous GR or PR was immunoprecipitated from KB-3-1 cells. The MAST1 promoter region in the DNA eluted from immune-precipitates was amplified by PCR. For MAST1 promoter reporter assay, MAST1 promoter variants were transfected into KB-3-1 or A2780 cells under indicated conditions and the promoter reporter activity was measured using LightSwitch dual luciferase assay system (SwitchGear Genomics).

### Surface plasmon resonance (SPR)

To study cisplatin and GR interaction, 1 μM of recombinant human GR-LBD WT or CA mutants were coupled to CM5 sensor chip. Various concentrations of cisplatin were prepared in 0.01 M HEPES pH 7.4, 0.005% v/v Surfactant P20, and 0.15 M NaCl solution, and injected over GR-LBD at 30 μl/min speed for 3 m at 20 °C. Multiple-cycle kinetic analysis was performed to quantify interaction between cisplatin and GR-LBD variants. The raw sensorgrams were blank-subtracted and dissociation constant (*K*_d_) values were obtained using BIA Evaluation Software v2.1 (GE Healthcare).

### Differential scanning fluorimetry and cellular thermal shift assay

Differential scanning fluorimetry (DSF) was performed by incubating 10 μM of recombinant GR with 0, 10, 20, or 30 μM of cisplatin for 10 m at 20 °C. GR-cisplatin mixture was employed to thermal shift reactions. Fluorescence intensity was recorded by a real-time PCR system and the data were analyzed using Protein Thermal Shift^TM^ Software v1.3. For cellular thermal shift assay, KB-3-1 cells were treated with DMSO, cisplatin or dexamethasone for 24 h. Collected cells were applied to 3 freeze-thaw cycles in PBS. A series of cell aliquots were heated at 50, 53, 55, 58, 61, 64, 67, and 70 °C for 3 min. The GR in the cell lysates of each aliquot was quantified by western blot analysis^33,55^.

### Immunofluorescence microscopy

KB-3-1 cells seeded on glass coverslips were immersed in PHEMO buffer (68 mM PIPES, 25 mM HEPES, 15 mM EGTA, and 3 mM MgCl_2_) with 0.05% glutaraldehyde, 3.7% formaldehyde, and 0.5% Triton X-100, and blocked in PBS containing 5% goat serum^46^. Cells were then incubated with GR antibody in PBS containing 2.5% goat serum followed by incubation with Alexa Fluor 488 conjugated goat anti-rabbit IgG. Samples were washed and mounted with antifade mounting medium with DAPI. Images were taken using Leica SP8 confocal microscope.

### Immunohistochemistry staining

Paraffin-embedded paired tumor specimens collected from head and neck cancer patients before and after receiving platinum-based chemotherapy were obtained from the Head and Neck Satellite Tissue Bank of Emory University. The study in human specimens was approved by the Institutional Review Board at Emory University. The study was conducted in compliance with ethical standards and good clinical practice. Clinical samples were collected with informed consent of the individuals or their guardians under Health Insurance Portability and Accountability Act (HIPAA) guidelines. IHC staining was conducted as previously described^33,55^. In brief, GR and MAST1 staining was performed by incubating the sections with GR antibody (1:400 dilution) and MAST1 antibody (1:200). Tumors collected from KB-3-1 or patient-derived xenograft (PDX) mice were stained with Ki-67 antibody (1:1000). The final immunoreactive score was determined by weighted index (WI) multiplying the intensity (0–3+) and extent of positivity scores (%) of stained cells^56^.

### In vivo studies and cytokine profiling

Animal studies were performed according to protocols reviewed and approved by the Institutional Animal Care and Use Committee of Emory University. Nude mice (Hsd:Athymic Nude-Foxn1^nu^, female, 6-week-old, Envigo) were injected with 0.5 × 10^6^ KB-3-1 cells. For in vivo model of cisplatin-resistant cancer, nude mice bearing KB-3-1 xenografts were administered with a sub-effective dose (0.5 mg/kg) and a higher dose (5 mg/kg) on day 5 and 10, respectively. For GR functional studies, cisplatin (2 or 4 mg/kg) were administered two times a week by intraperitoneal (i.p.) injection when tumor sizes reached up to 100 mm^3^. For PDX studies, head and neck cancer patient tumor was implanted into NOD scid gamma mice (female, 6-week-old, Jackson Laboratory). Approximately 1500 mm^3^ sized tumors were excised, evenly diced, and implanted in the flank of nude mice. Ovarian cancer PDX model was obtained from Jacksons Laboratory. The mice were randomly divided into groups when the tumor size reached 100 mm^3^. For syngeneic mouse model, luciferase gene was transduced into murine ovarian ID8 cells and injected into C57BL/6 mice (female, 6-week-old, Envigo) by intraperitoneal (i.p.) injection. Cisplatin (5 mg/kg) and dexamethasone (0.1 mg/kg) were given by i.p. injection twice a week. Lestaurtinib (20 mg/kg/day) was administered by subcutaneous injection. KB-3-1 or PDX tumors were measured and volumes were calculated as 4*π*/3 x (width/2)^2^ x (length/2). Blinding and allocation concealment were used. For syngeneic ID8 mouse model, tumor growth was monitored by bioluminescence imaging (BLI) analysis as described^57^. Blood was harvested at the experimental endpoint and mouse inflammatory cytokines, including IL-6, IL-23, IL-1α, TNF-α, MCP-1, GM-CSF, IFN-β, IFN-γ, IL-17, IL-12p70, IL-27, IL-10, and IL-1β in plasma were quantified by flow cytometry analysis. IL-1β was not detected in the C57BL/6 mice plasma.

### Statistical analysis

Statistical analyses were performed using GraphPad Prism 9.0. Sample size was not predetermined by statistics. One representative experiment of multiple experiments is shown for each immunoblotting figure panel. Error bars represent mean ± standard deviation (SD), except tumor growth curves and blot density analysis in Fig. 6a, which denote mean ± standard error of the mean (SEM). Statistical significance was based on two-tailed Student’s *t*-test for Figs. 1a bottom, 1d, 1f, 2h, and 3e, Pearson’s correlation coefficient for 3g, and one-way or two-way ANOVA for all other data of multiple-comparisons. Statistical analyses are based on assumptions of normal distribution and homogeneity of variances. *P*-values of < 0.05 were regarded as statistically significant.

### Reporting summary

Further information on research design is available in the Nature Research Reporting Summary linked to this article.

## Supplementary information

Supplementary Information

Reporting Summary

## Data Availability

The RNA-seq datasets generated in the course of this study are available on the NCBI GEO database under the accession number GSE179263 Publicly available datasets obtained from http://firebrowse.org/?cohort=OV and https://string-db.org/ were used in the study. Source data are provided with this paper.

## Source data

Source Data
