# Supplementary material for: Cisplatin-mediated activation of glucocorticoid receptor induces platinum resistance via MAST1
Source: Nat Commun. 2021 Aug 16;12:4960. doi: 10.1038/s41467-021-24845-8 (PMC8368102; doi:10.1038/s41467-021-24845-8)
Supplement: Supplementary file 1 — Supplementary Information [file 41467_2021_24845_MOESM1_ESM.pdf]

Cisplatin-mediated activation of glucocorticoid receptor induces platinum resistance *via* MAST1

Pan et al. - Supplementary Figures and Table

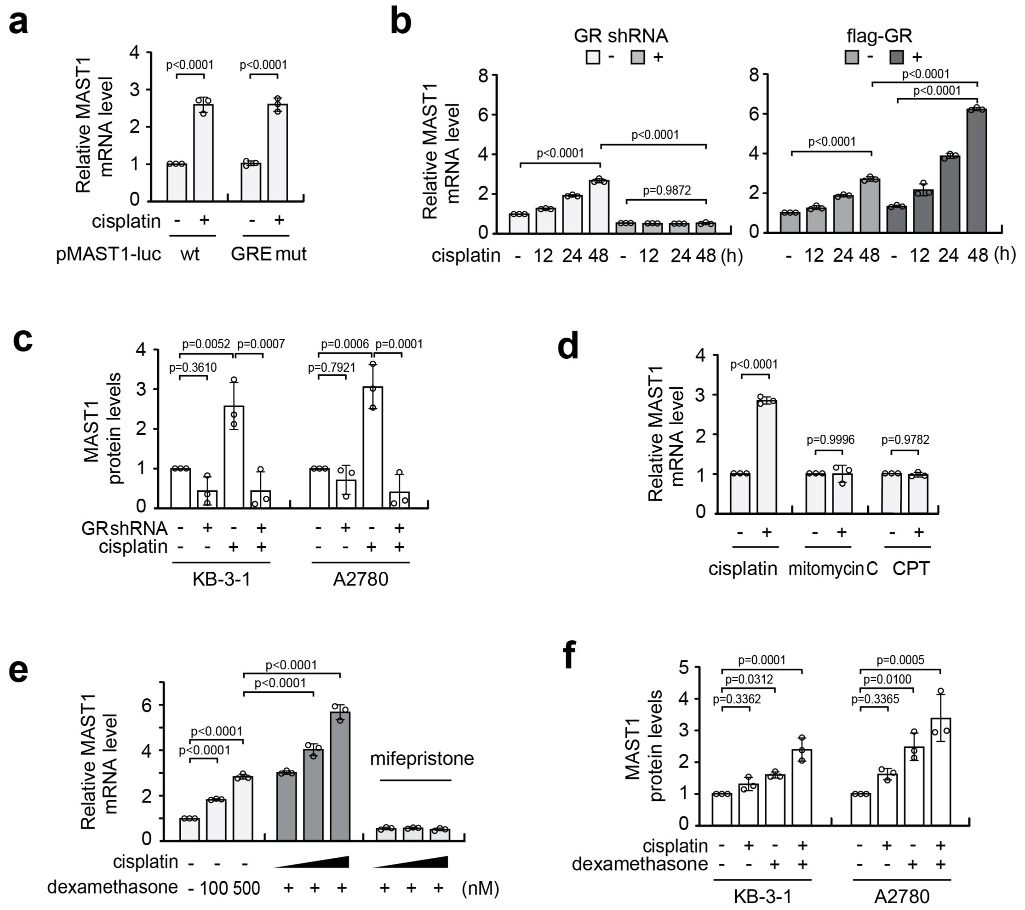

Supplementary Figure 1. The transcription factor GR induces MAST1 expression. (a) Total cellular MAST1 mRNA levels in cells expressing WT or GRE mutated MAST1 promoter reporter. **(b)** MAST1 mRNA level in KB-3-1 cells with GR knockdown or overexpression and cisplatin treatment. **(c)** Effect of GR knockdown on MAST1 protein level in the presence or absence of cisplatin. **(d)** Effect of chemotherapy agents including cisplatin, mitomycin C, and camptothecin (CPT) on MAST1 mRNA level. **(e)** MAST1 mRNA level in KB-3-1 cells treated with increasing concentrations of cisplatin (0.1, 0.2, 0.5 μg/ml) and dexamethasone (100 and 500 nM). **(f)** Induction of MAST1 protein by cisplatin and dexamethasone. Densitometry analyses of three independent biological replicates of MAST1 blots are shown in panels (c) and (f), and the representative blot images are shown in Figures 2g and 2k, respectively. Experimental conditions for (a), (b), (d) and (e) are same as described in Figures 2e, 2f, 2i, and 2j, respectively. Data are mean ± SD from three independent biological experiments. Statistical analysis was performed by 1-way ANOVA. Source data are provided as a Source Data file.

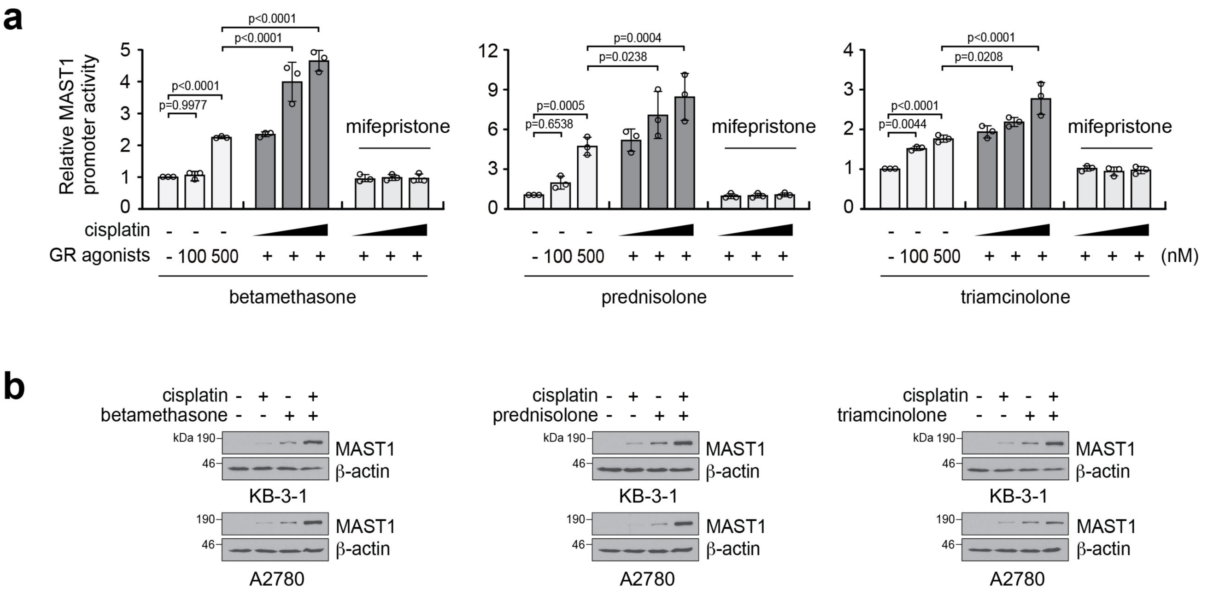

Supplementary Figure 2. Cisplatin and GR agonists induce MAST1 expression. **(a)** KB-3-1 cells were treated with cisplatin (0.1, 0.2, 0.5 μg/ml) and GR agonists betamethasone, prednisolone, or triamcinolone (100, 500 nM) for MAST1 promoter activity assay. 10 μM of mifepristone, which is a GR antagonist, was used as a control. **(b)** KB-3-1 and A2780 cells were treated with cisplatin (1 μg/ml), GR agonists betamethasone, prednisolone, or triamcinolone (500 nM), or combination and MAST1 protein levels were assessed by immunoblotting. Uncropped blots are in Source Data. Data are mean ± SD from three independent biological replicates for (a) and representative of three independent biological experiments for (b). Statistical analysis was performed by 1-way ANOVA. Source data are provided as a Source Data file.

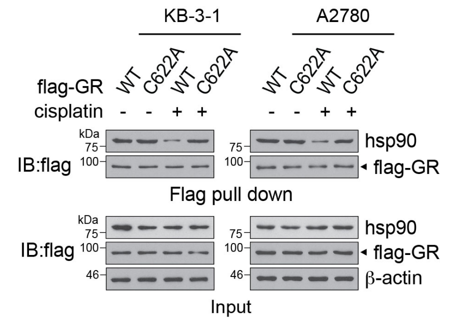

Supplementary Figure 3. GR WT but not C622A mutant dissociates from hsp90 upon cisplatin treatment. Flag tagged wild type (WT) or C622A mutant form of GR was overexpressed in KB-3-1 and A2780 cells with endogenous GR knockdown. Cells were treated with 1μg/ml cisplatin for 24 h followed by flag pull down. Flag-GR WT or C622A-bound hsp90 in the presence or absence of cisplatin was assessed by immunoblotting. Data are from one biological experiment. Uncropped blots are in Source Data.

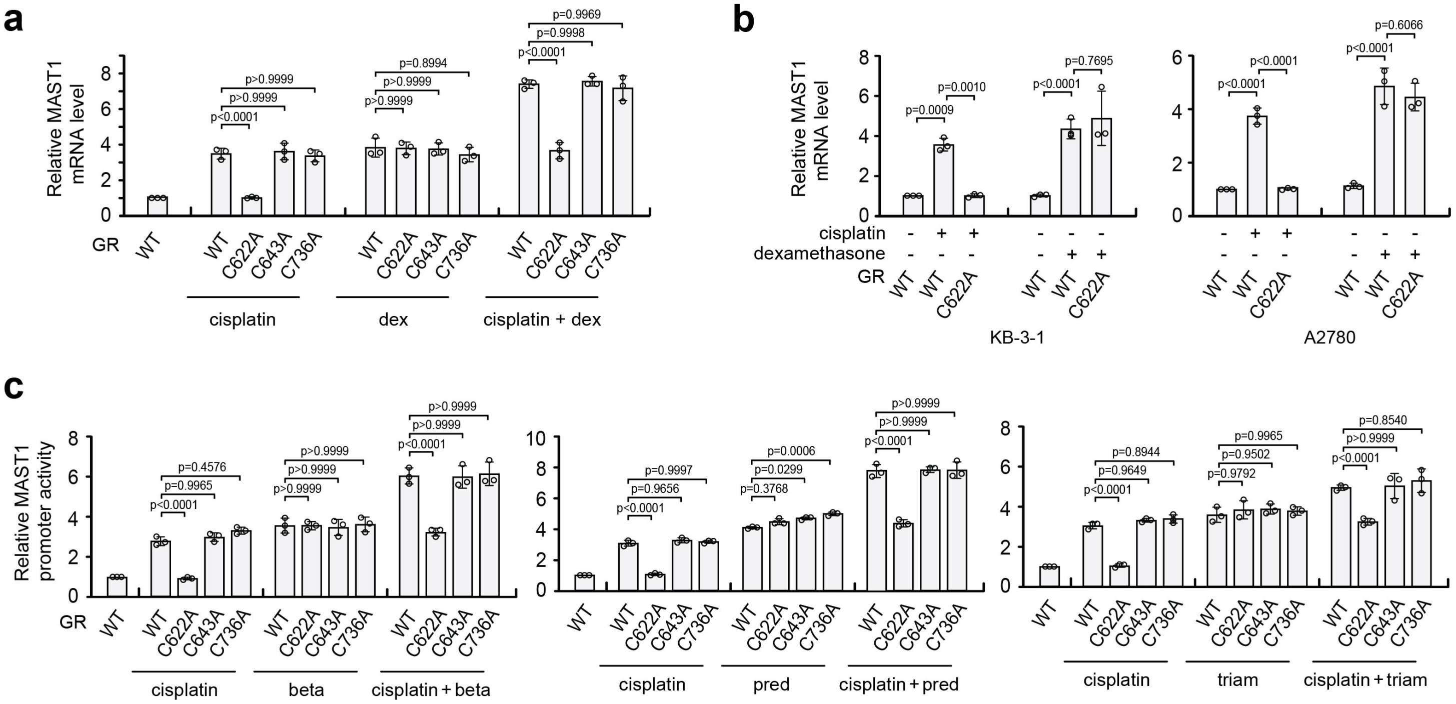

Supplementary Figure 4. Cisplatin but not GR agonists mediates MAST1 transcription through GR C622. (a and b) Effect of cisplatin and dexamethasone on MAST1 gene expression in KB-3-1 expressing GR WT or CA mutants (a) or in KB-3-1 and A2780 cells expressing GR WT or C622A mutant (b). Cells were overexpressed with GR variants and treated with cisplatin and dexamethasone as described in Figures 2h and 2i, respectively. **(c)** Effect of cisplatin and GR agonists on MAST1 promoter activity in cells expressing GR WT or CA mutants. KB-3-1 cells were treated with 1μg/ml cisplatin or 500 nM betamethasone (beta), prednisolone (pred), or triamcinolone (triam) for 24 h followed by MAST1 promoter activity assay. Data are mean ± SD from three independent biological replicates. Statistical analysis was performed by 1-way ANOVA. Source data are provided as a Source Data file.

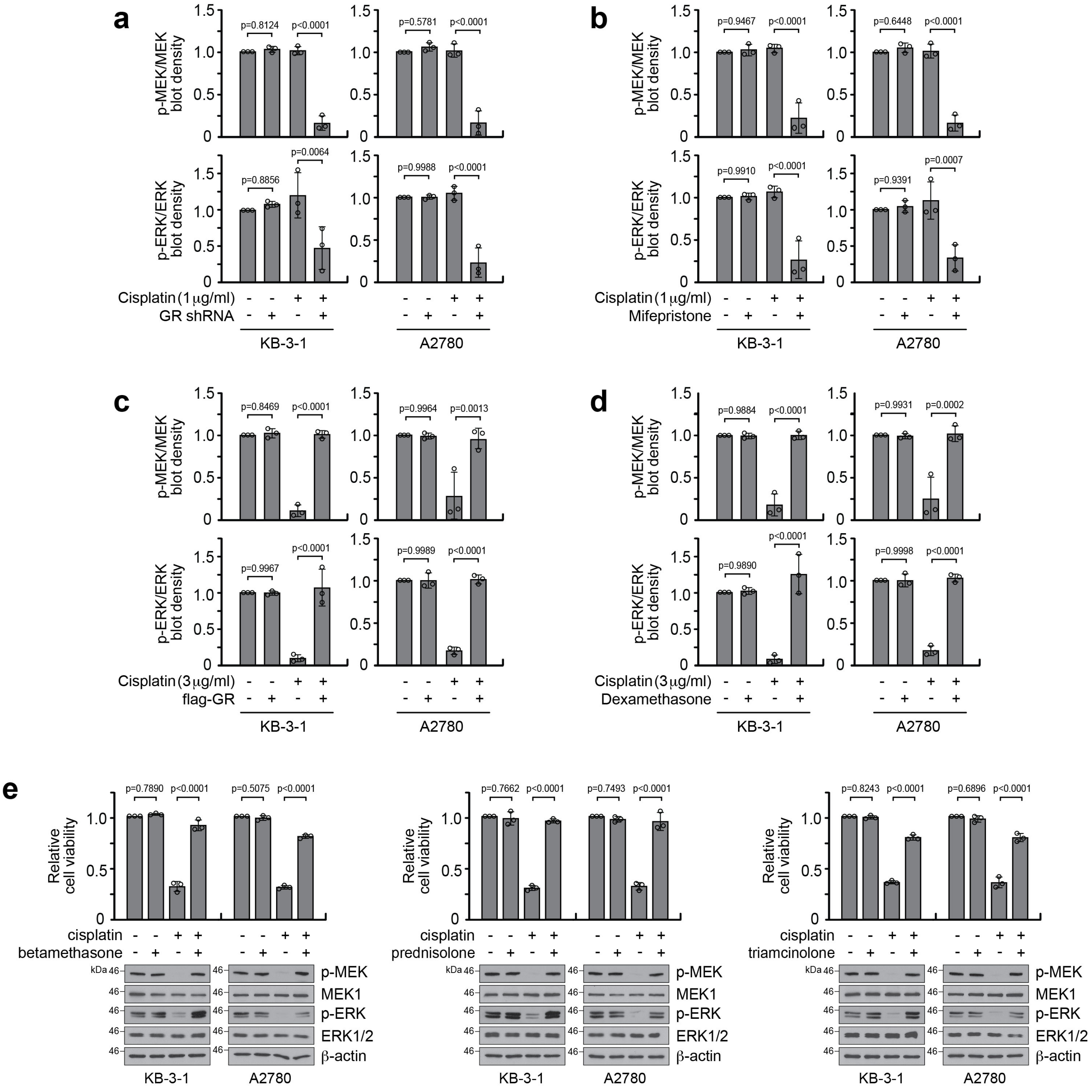

Supplementary Figure 5. GR confers cisplatin resistance through MEK-ERK activation. (a-d) Densitometry analyses of three independent biological replicates of phospho-MEK/MEK and phospho-ERK/ERK blots in KB-3-1 and A2780 cells with GR modulation are shown and the representative blot images are shown in Figures 5a-5d, respectively. **(e)** Cell viability and MEK/ERK activation in GR enhanced KB-3-1 and A2780 cells by 500 nM of betamethasone, prednisolone, or triamcinolone in the presence and absence of 3 μg/ml of cisplatin. Uncropped blots are in Source Data. Data are mean ± SD from three independent biological replicates for (a-e). Statistical analysis was performed by 1-way ANOVA. Source data are provided as a Source Data file.

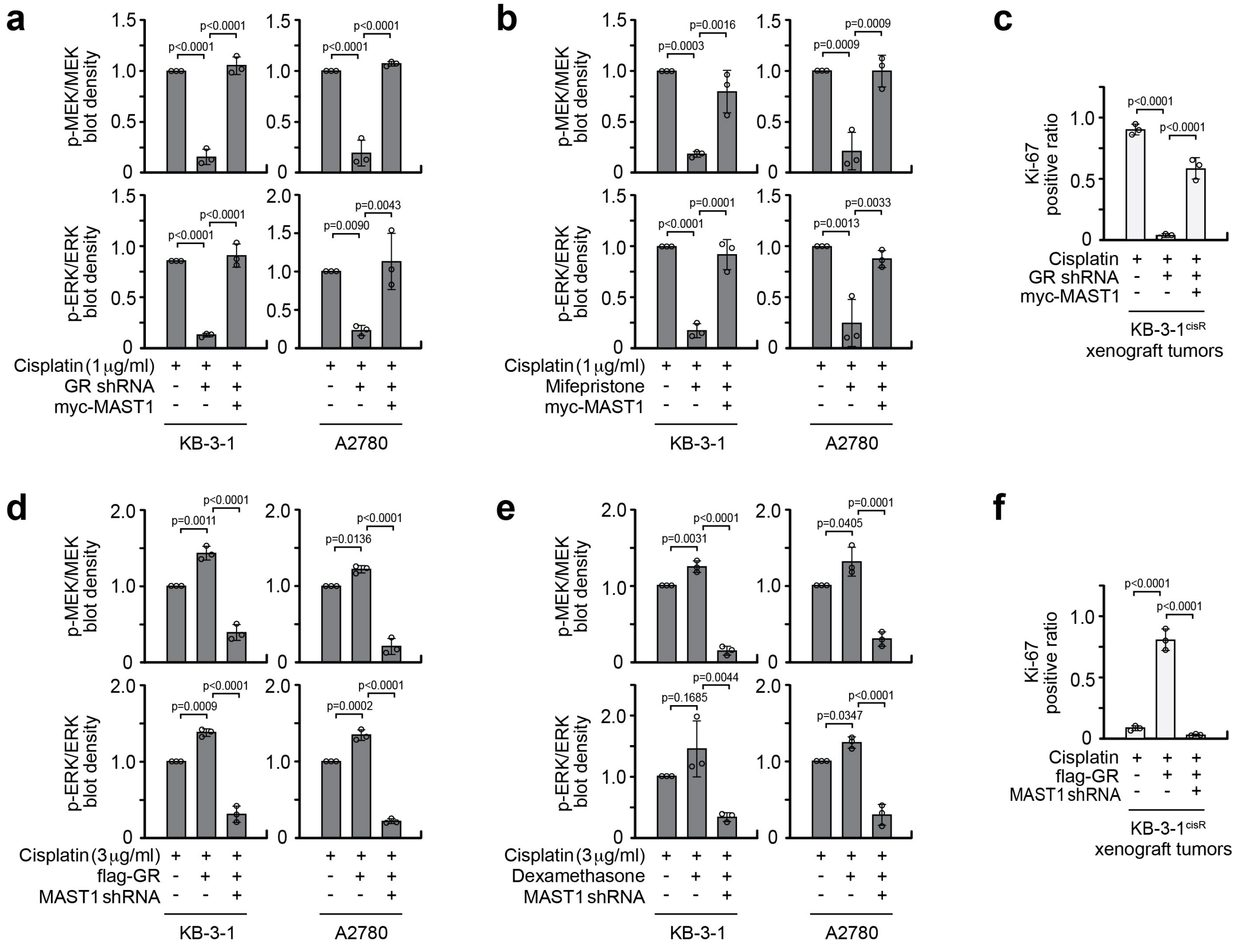

Supplementary Figure 6. GR-MAST1 confers MEK-ERK activation and cisplatin resistance. (a-b and d-e) Densitometry analyses of three independent biological replicates of phospho-MEK/MEK and phospho-ERK/ERK blots in KB-3-1 and A2780 cells with GR and MAST1 modulations are shown and the representative blot images are shown in Figures 7a, 7b, 7f, and 7g, respectively. **(c and f)** Tumor cells positive for Ki-67 were quantified for Figures 7d and 7i. One thousand cells per mouse were analyzed using ImageJ software and the representative images are shown in Figures 7d and 7i, respectively. Data are mean ± SD from three independent biological replicates. Statistical analysis was performed by 1-way ANOVA. Source data are provided as a Source Data file.

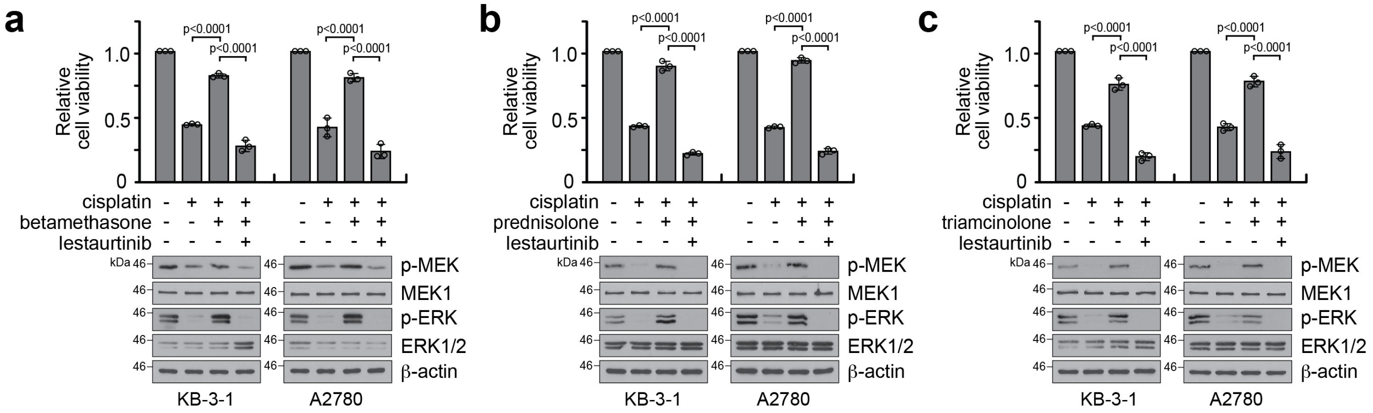

Supplementary Figure 7. Lestaurtinib suppresses MAST1-MEK/ERK activation and cisplatin resistance mediated by GR agonists. Effect of lestaurtinib on cell viability and MAPK activity in cisplatin and betamethasone (a), prednisolone (b), or triamcinolone (c) co-treated KB-3-1 and A2780 cells. Cells were treated with 100 nM lestaurtinib, 500 nM GR agonist, and 3 μg/ml cisplatin. Uncropped blots are in Source Data. Data are mean ± SD from three independent biological replicates. Statistical analysis was performed by 1-way ANOVA. Source data are provided as a Source Data file.

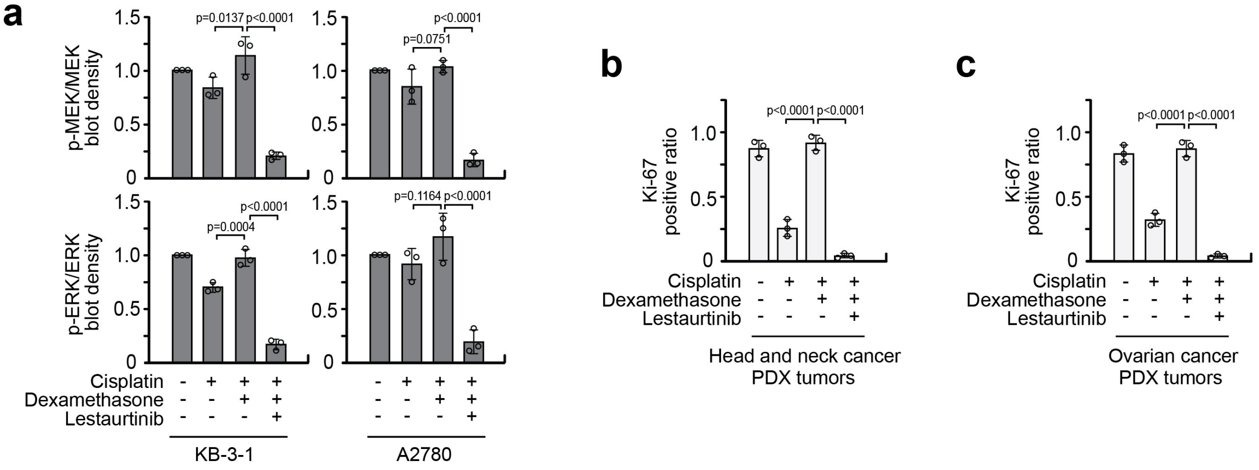

Supplementary Figure 8. Lestaurtinib inhibits MEK-ERK activity and cisplatin resistance induced by dexamethasone. (a) Densitometry analyses of three independent biological replicates of phospho-MEK/MEK and phospho-ERK/ERK blots in KB-3-1 and A2780 cells treated with cisplatin (3 μg/ml), dexamethasone (500 nM), and lestaurtinib (100 nM); representative blot images are shown in Figure 8a. **(b and c)** Ki-67 positive tumor cells among 1000 cells/mouse were quantified for Figures 8c and 8d by ImageJ software. Representative images of (b) and (c) are shown in Figures 8c and 8d, respectively. Data are mean ± SD from three independent biological replicates. Statistical analysis was performed by 1-way ANOVA. Source data are provided as a Source Data file.

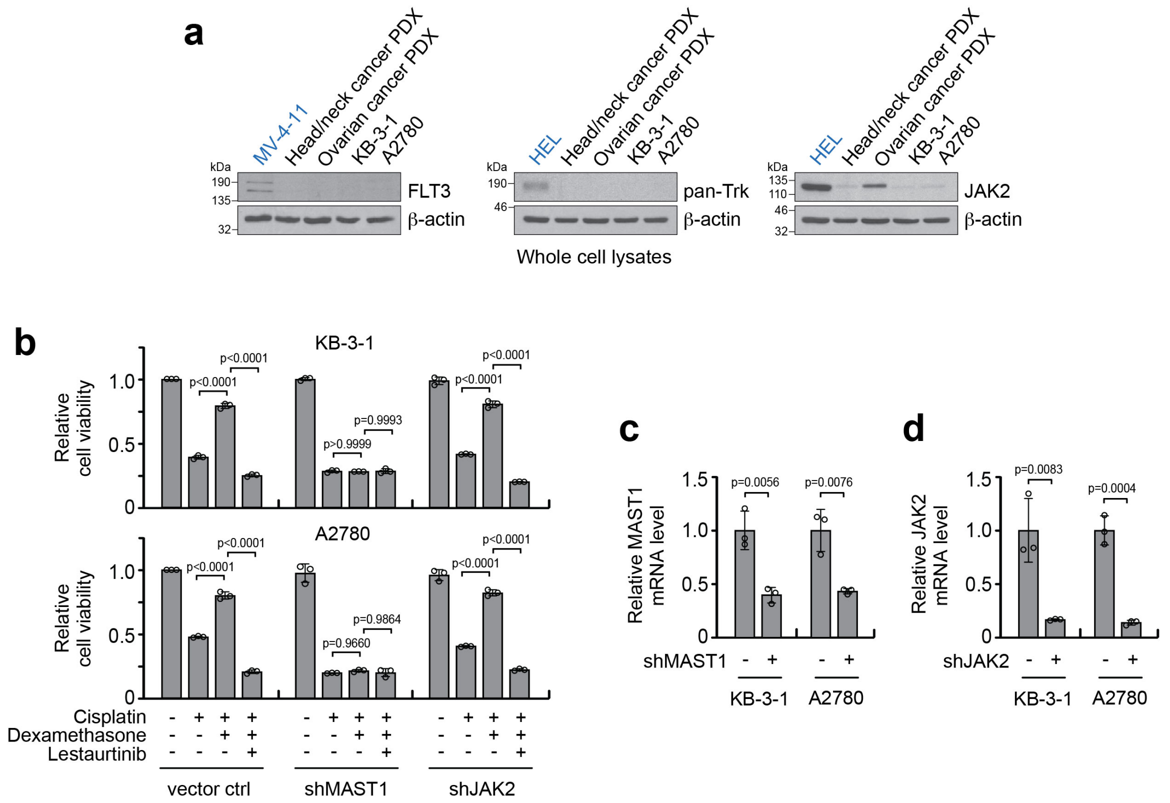

Supplementary Figure 9. Lestaurtinib sensitizes cells to cisplatin treatment by inhibiting MAST1. (a) FLT3, Trk, and JAK2 expression levels in cancer cells used in the study. Leukemia HEL cells and MV-4-11 cells were used as positive controls. Uncropped blots are in Source Data. **(b)** Effect of MAST1 or JAK2 downregulation on cisplatin sensitivity induced by lestaurtinib. KB-3-1 and A2780 cells with empty vector, MAST1 shRNA, or JAK2 shRNA clones were treated with lestaurtinib (100 nM), dexamethasone (500 nM) and cisplatin (3 μg/ml) for 48 h and cell viability was determined by CellTiter-Glo Luminescent Viability assay. **(c and d)** Knockdown of MAST1 (c) and JAK2 (d) was determined by quantitative RT-PCR. Data are mean ± SD from three independent biological replicates for (b-d) and one biological experiment for (a). Statistical analysis was performed by 1-way ANOVA for (b) and unpaired 2-tailed student's *t* test for (c) and (d). Source data are provided as a Source Data file.

Name	Sequence
GR pENTR clone Forward	CACCATGGACTACAAGGACGACGACGACAAGGACTCCAAAGAA TCATTAACTCCT
GR pENTR clone Reverse	TCACTTTTGATGAAACAGAAG
PR pENTR clone Forward	CACCATGGACTACAAGGACGACGACGACAAGACTGAGCTGAA GGCAAAGGGTCCC
GR pENTR clone Reverse	TCACTTTTTATGAAAGAGAAG
GR-LBD pENTR clone Forward	CACCATGCAACTCACCCCTACCCTGGTG
GR-LBD pENTR clone Reverse	TCACTTTTGATGAAACAGAAG
GR-LBD F602S A605V mutant Forward	TACTCCTGGATGTCTCTTATGGTATTTGCTCTGGGG
GR-LBD F602S A605V mutant Reverse	CCCCAGAGCAAATACCATAAGAGACATCCAGGAGTA
GR-LBD V702A E705G mutant Forward	GGAAAAGCCATTGACAAGAGGGGAGGAAACTCCAGC
GR-LBD V702A E705G mutant Reverse	GCTGGAGTTTCCTCCCCTCTTGTCAATGGCTTTTCC
GR-LBD M752T mutant Forward	ATTGAATTCCCCGAGACGTTAGCTGAAATCATC
GR-LBD M752T mutant Reverse	GATGATTTCAGCTAACGTCTCGGGGAATTCAAT
GR-LBD C622A mutant Forward	GTGCAAACCTGCTGGCTTTTGCTCCTGATCTG
GR-LBD C622A mutant Reverse	CAGATCAGGAGCAAAAGCCAGCAGGTTTGCAC
GR-LBD C643A mutant Forward	CTGCATGTACGACCAAGCTAACACATGCTGTATG
GR-LBD C643A mutant Reverse	CATACAGCATGTGTTAGCTTGGTCGTACATGCAG
GR-LBD C736A mutant Forward	AATCTCCTTAACTATGCCTTCCAAACATTTTTG
GR-LBD C736A mutant Reverse	CAAAAATGTTTGGAAGGCATAGTTAAGGAGATT
MAST1 promoter GRE binding mutant Forward	CCCGGCGGCAGTAACCCTGGCCGCACCAAGAG
MAST1 promoter GRE binding mutant Reverse	CTCTTGGTGCGGCCAGGGTTACTGCCGCCGGG

Supplementary Table 1. Names and sequences of the primers used in the study.
