# Supplementary material for: Cisplatin-mediated activation of glucocorticoid receptor induces platinum resistance via MAST1
Source: Nat Commun. 2021 Aug 16;12:4960. doi: 10.1038/s41467-021-24845-8 (PMC8368102; doi:10.1038/s41467-021-24845-8)
Supplement: Supplementary file 2 — Reporting Summary [file 41467_2021_24845_MOESM2_ESM.pdf]

nature research

Corresponding author(s): Sumin Kang

Last updated by author(s): June 10, 2021

Reporting Summary

Nature Research wishes to improve the reproducibility of the work that we publish. This form provides structure for consistency and transparency in reporting. For further information on Nature Research policies, see our Editorial Policies and the Editorial Policy Checklist.

Statistics

For all statistical analyses, confirm that the following items are present in the figure legend, table legend, main text, or Methods section.

n/a	Confirmed	
☐	☒	The exact sample size (*n*) for each experimental group/condition, given as a discrete number and unit of measurement
☐	☒	A statement on whether measurements were taken from distinct samples or whether the same sample was measured repeatedly
☐	☒	The statistical test(s) used AND whether they are one- or two-sided<br>*Only common tests should be described solely by name; describe more complex techniques in the Methods section.*
☒	☐	A description of all covariates tested
☒	☐	A description of any assumptions or corrections, such as tests of normality and adjustment for multiple comparisons
☐	☒	A full description of the statistical parameters including central tendency (e.g. means) or other basic estimates (e.g. regression coefficient) AND variation (e.g. standard deviation) or associated estimates of uncertainty (e.g. confidence intervals)
☐	☒	For null hypothesis testing, the test statistic (e.g. *F*, *t*, *r*) with confidence intervals, effect sizes, degrees of freedom and *P* value noted<br>*Give P values as exact values whenever suitable.*
☒	☐	For Bayesian analysis, information on the choice of priors and Markov chain Monte Carlo settings
☒	☐	For hierarchical and complex designs, identification of the appropriate level for tests and full reporting of outcomes
☐	☒	Estimates of effect sizes (e.g. Cohen's *d*, Pearson's *r*), indicating how they were calculated

Our web collection on statistics for biologists contains articles on many of the points above.

Software and code

Policy information about availability of computer code

Data collection	Data were collected using BIA Evaluation v2.1, Protein Thermal Shift v1.3, and ImageJ softwares.
Data analysis	GraphPad Prism 9.0 was used for data analysis throughout the manuscript .

For manuscripts utilizing custom algorithms or software that are central to the research but not yet described in published literature, software must be made available to editors and reviewers. We strongly encourage code deposition in a community repository (e.g. GitHub). See the Nature Research guidelines for submitting code & software for further information.

Data

Policy information about availability of data

All manuscripts must include a data availability statement. This statement should provide the following information, where applicable:

- Accession codes, unique identifiers, or web links for publicly available datasets
- A list of figures that have associated raw data
- A description of any restrictions on data availability

The RNA-seq data that generated in this study have been deposited in figshare with the identifier https://doi.org/10.6084/m9.figshare.14511465.v1. Publicly available datasets obtained from http://firebrowse.org/ and https://string-db.org/ were used in the study. Source data are provided with this paper.

Field-specific reporting

Life sciences study design

All studies must disclose on these points even when the disclosure is negative.

Sample size	For in vivo experiments, a sample size of n=8-10 mice was used per experimental group. Sample size was determined based on our previous experience and published data (Cancer Cell 2018; PMID 30033091), which was sufficient to produce statistically significant results. No statistical method was used to predetermine sample size. For in vitro study, three biological replicates were considered for most the experiments. Exceptions were made when the data were validated by alternative methods (e.g. fig 4d) or sample access was limited (e.g. supplementary fig 9a).
Data exclusions	No data were excluded.
Replication	Mechanistic studies are from three independent biological replicates. For animal studies, 8 mice/group (Figures 7c, d, h, i, and 8c), 9 mice/group (Figures 1a and 8d), and 10 mice/group (Figures 8e, f) were used. All attempts at replication were successful for these experiments.
Randomization	In our experimental design, we ensured that equal numbers of animals (in vivo) or cells (in vitro) were randomly assigned to each group and subjected to each treatment that we tested.
Blinding	The Investigators were not blinded to allocation during experiments. Because the reported outcomes are based on non-subjective measurements. Studies were designed so that multiple samples were arranged and measured side-by-side in a highly consistent manner. Appropriate controls were included for every study design.

Reporting for specific materials, systems and methods

We require information from authors about some types of materials, experimental systems and methods used in many studies. Here, indicate whether each material, system or method listed is relevant to your study. If you are not sure if a list item applies to your research, read the appropriate section before selecting a response.

Materials & experimental systems

n/a	Involved in the study	
☐	☒	Antibodies
☐	☒	Eukaryotic cell lines
☒	☐	Palaeontology and archaeology
☐	☒	Animals and other organisms
☐	☒	Human research participants
☒	☐	Clinical data
☒	☐	Dual use research of concern

Methods

n/a	Involved in the study	
☒	☐	ChIP-seq
☒	☐	Flow cytometry
☒	☐	MRI-based neuroimaging

Antibodies

Antibodies used	Antibodies against GR (12041/D6H2L), PR (3153/C89F7), phospho-MEK1/2 (S221) (2338/166F8), MEK1/2 (9126/47E6), phospho-ERK1/2 (T202/Y204) (4376/20G11), ERK1/2 (4695/137F5), myc (2276/9B11), hsp90 (4877/C45G5), phospho-histone H2A.X (S139) (9718/20E3), PARP (9532/46D11), pan-Trk (92991/A7H6R), and Jak2 (3230/D2E12) were obtained from Cell Signaling Technology. MAST1 antibodies (NBP2-17228 and NBP1-81453) were obtained from Novus Biologicals. Antibodies against FLAG (F7425 and F1804/M2) and beta-actin (A1978/AC-15) were purchased from Sigma-Aldrich. Antibodies against cisplatin-modified DNA (ab103261/CP9/19) and Ki-67 (ab92742/EPR3610) were obtained from Abcam. Anti-ATF-2 antibody (sc-242/F2BR-1) and anti-FLT3 (sc-479/C-20) antibody were from Santa Cruz Biotechnology. Antibodies against CEBPZ (31-163) and NFATC1 (MA3-024/7A6) were purchased from ProSci and Invitrogen, respectively. Anti-CAR/NR1I3 antibody (PP-N4111-00/N4111) was obtained from R&D Systems. Anti-Rabbit IgG (H+L) cross-adsorbed secondary antibody-Alexa Fluor 488 (A-11008/RRID AB_143165), anti-rabbit IgG (H+L) secondary antibody-HRP (31460/RRID AB_228341), and anti-mouse IgG (H+L) secondary antibody-HRP (31430, RRID AB_228307) were obtained from Invitrogen. 1 to 1000 dilution was used for all the primary antibodies, and 1 to 2000 dilution was used for all the secondary antibodies.
Validation	All primary antibodies used in this study were commercially available and validated by the respective manufacturer for their use in Western Biotting, immunofluorescence staining, and immunohistochemistry staining to detect target proteins in human cells. Additional validation was performed prior to the study using positive control cells that express target proteins or negative control cells lacking target protein by genetic manipulation.

Eukaryotic cell lines

Policy information about cell lines

Cell line source(s)	A2780, ID8, and KB-3-1 cells were purchased from Sigma Aldrich. KB-3-1 is a derivative of HeLa commonly used in multiple drug resistance studies. 293T cells were from American Type Culture Collection. Ovarian PDX tumors for organoid culture were purchased from Jackson Laboratory.

Authentication	All cell lines were authenticated by STR profiling.
Mycoplasma contamination	All cell lines were tested negative for mycoplasma contamination.
Commonly misidentified lines (See ICLAC register)	No commonly misidentified cell lines were used in this study.

Animals and other organisms

Policy information about studies involving animals; ARRIVE guidelines recommended for reporting animal research

Laboratory animals	Nude mice (Hsd:Athymic Nude-Foxn1nu, female, 6-week-old, Envigo); C57BL/6J mice (female, 6-week-old, Envigo); NOD scid gamma mice (NOD.Cg-Prkdc scid Il2rg tm1Wjl/SzJ, female, 6-week-old, Jackson Laboratory)
Wild animals	Wild animals were not used.
Field-collected samples	Field-collected samples were not used.
Ethics oversight	Ethical approval was provided by the IACUC office at Emory University.

Note that full information on the approval of the study protocol must also be provided in the manuscript.

Human research participants

Policy information about studies involving human research participants

Population characteristics	*Describe the covariate-relevant population characteristics of the human research participants (e.g. age, gender, genotypic information, past and current diagnosis and treatment categories). If you filled out the behavioural & social sciences study design questions and have nothing to add here, write "See above."*
Recruitment	*Describe how participants were recruited. Outline any potential self-selection bias or other biases that may be present and how these are likely to impact results.*
Ethics oversight	*Identify the organization(s) that approved the study protocol.*

Note that full information on the approval of the study protocol must also be provided in the manuscript.
